# Artificial intelligence for monitoring hand hygiene compliance in healthcare settings: A scoping review

**DOI:** 10.1371/journal.pone.0347683

**Published:** 2026-04-21

**Authors:** Xinran Lin, Yu Lv, Qian Xiang, Minhong Cai, Pingping Wang

**Affiliations:** 1 School of Medicine, University of Electronic Science and Technology of China, Chengdu, Sichuan, China; 2 Public Health Department, Sichuan Provincial People’s Hospital, School of Medicine, University of Electronic Science and Technology of China, Chengdu, Sichuan, China; 3 Healthcare-Associated Infection Control Center, Sichuan Provincial People’s Hospital, School of Medicine, University of Electronic Science and Technology of China, Chengdu, Sichuan, China; Ege University, Faculty of Medicine, TÜRKIYE

## Abstract

**Background:**

Hand hygiene is a fundamental measure for preventing healthcare-associated infections, yet traditional monitoring methods are significantly limited by the Hawthorne effect, high resource demands, and an inability to assess procedural quality. Artificial intelligence (AI) technology has emerged as a transformative, automated, and objective approach to address these long-standing challenges.

**Objective:**

This scoping review sought to systematically map the existing evidence, technical pathways, performance metrics, and implementation challenges of AI for monitoring hand hygiene compliance in healthcare settings.

**Methods:**

Following the Joanna Briggs Institute (JBI) methodological framework and PRISMA-ScR guidelines, we searched five major databases (PubMed, Scopus, Embase, Web of Science, and IEEE Xplore) for articles published between January 2000 and September 2025, supplemented by grey literature searching and backward citation tracking. Two reviewers independently screened records, assessed full-text reports for eligibility, and extracted data, which were synthesized using descriptive statistics and thematic analysis.

**Results:**

Of 800 records identified through database and supplementary searches, 45 studies (2007–2025) were included. The primary technical pathways identified were computer vision (53.3%), wearable sensors (24.4%), Internet of Things-integrated systems (13.3%), and radar/radio frequency-based systems (8.9%). While computer vision achieved high accuracy (95%) in setting-specific ICU models, performance dropped to 56% in generalizable models. Wearable systems demonstrated portability but showed 5%–10% lower specificity than vision-based approaches. Most evidence is derived from small-scale technical validations, with a significant lack of formal fairness analysis and evaluation of clinical workflows or cost-effectiveness.

**Conclusion:**

AI-based hand hygiene monitoring shows promise for supporting more objective and scalable hand hygiene surveillance in healthcare settings. However, the field remains at a largely pre-translational stage. Future research should shift from technical feasibility toward implementation science, focusing on establishing standardized motion databases, evaluating ethical governance (e.g., privacy and automation bias), and conducting pragmatic trials to demonstrate sustained clinical benefit and organizational sustainability.

## 1 Introduction

Hand hygiene is widely recognized as one of the most fundamental, effective, and cost‐effective measures for preventing healthcare‐associated infections [1–5]. These infections significantly contribute to patient morbidity and mortality while imposing substantial economic burdens on healthcare systems [6–9]. In 2009, the World Health Organization (WHO) issued the Guidelines on Hand Hygiene in Health Care, introducing the “Five Moments for Hand Hygiene” as a foundational framework for clinical practice [1]. Despite decades of global efforts, compliance with hand hygiene protocols among healthcare workers (HCWs) remains suboptimal [10–12]. A retrospective analysis conducted by the WHO reported that the average baseline compliance rate among HCWs was merely 38.7%, with striking variability across different clinical contexts (ranging from 5% to 89%) [1,13]. Such disparities may reflect true heterogeneity in hand hygiene practices or may arise from methodological inconsistencies in compliance monitoring across institutions.

Current monitoring of hand hygiene compliance relies primarily on direct observation, self-reporting, and the measurement of hand hygiene product consumption [14–18]. However, these approaches are hampered by inherent and substantial limitations. Direct observation, considered the gold standard [1,16], is undermined by the Hawthorne effect, where awareness of monitoring alters behavior [19–21]. Additionally, it demands significant resources, yields limited sample representativeness, and is susceptible to observer subjectivity [22–24]. Self-reported data are significantly influenced by social desirability bias, leading to overestimation of true compliance rates [1]. Product consumption, used as a proxy measure, fails to accurately reflect compliance quality or appropriateness, as key contextual factors such as patient load and specific care activities are not incorporated [25,26]. These methodological limitations have significantly impeded the acquisition of accurate and objective baseline data on hand hygiene compliance.

The rapid advancement of artificial intelligence (AI), particularly in deep learning and computer vision, has opened transformative avenues for addressing this long-standing challenge [27–29]. AI-driven automated monitoring systems enable continuous, objective, and contactless data collection on hand hygiene behaviors through cameras or sensors installed in clinical environments [30–32]. Using deep learning algorithms, hand hygiene actions performed by HCWs during room entry and exit can be automatically identified, allowing for large-scale, around-the-clock monitoring [33,34]. Compared with direct observation, AI-based monitoring may reduce Hawthorne bias by providing a less intrusive means of assessment [16,25], although awareness of being monitored may still influence behavior. In addition, real-time audiovisual feedback can be delivered to prompt non-compliant individuals, while aggregated data are leveraged for quality improvement and precision management [35].

The application of AI for hand hygiene monitoring has been explored in preliminary studies and commercial products, showing considerable promise. However, this field remains in its early developmental stage and is marked by highly fragmented evidence. Existing literature encompasses diverse technical approaches, including systems based on Red-Green-Blue cameras, depth sensors, and Internet of Things (IoT) frameworks. Varied algorithmic architectures have been implemented and evaluated across different clinical environments such as Intensive Care Units (ICUs), general wards, and operating rooms. A systematic synthesis of this heterogeneous body of evidence has not yet been conducted.

This study conducted a scoping review of the evidence on AI for monitoring hand hygiene compliance in healthcare settings. A scoping review was considered appropriate for mapping the breadth, concepts, and types of evidence in this emerging field and for identifying key elements and research gaps. The review systematically characterized AI technology types and features, summarized their implementation and performance across healthcare scenarios, examined technical, practical, and ethical challenges, and identified future research directions and clinical translation pathways based on the available evidence.

## 2 Materials and methods

This scoping review was conducted in accordance with the Joanna Briggs Institute (JBI) methodological framework [36]. The JBI framework was selected because this review aimed to map the breadth, characteristics, and research gaps of an emerging and heterogeneous literature rather than estimate pooled effectiveness, and because it provides a clear structure for defining population, concept, and context and for guiding study identification, selection, and synthesis. The review was reported following the Preferred Reporting Items for Systematic Reviews and Meta-Analyses Extension for Scoping Reviews (PRISMA-ScR) [37] (S1 Appendix). PRISMA-ScR was used to ensure transparent and complete reporting of the review process. The review protocol was prospectively registered with the Open Science Framework (https://osf.io/7csxk).

### 2.1 Research questions

This scoping review aimed to systematically characterize the current landscape of AI applications for monitoring hand hygiene compliance in healthcare settings. To address this aim, the review considered the following four research questions.

1. What technical approaches are currently being employed in AI systems designed for monitoring hand hygiene compliance?
2. How do these AI technologies perform in terms of key metrics, and what empirical evidence supports their practical effectiveness?
3. What limitations currently affect the implementation of AI technologies for hand hygiene compliance monitoring?
4. Based on existing evidence, what are the emerging research trends and potential pathways for clinical implementation in this field?

### 2.2 Eligibility criteria

Explicit inclusion and exclusion criteria were developed according to the PCC framework (Population, Concept, Context) from the JBI [36] (Table 1).

**Table 1:** Inclusion and exclusion criteria.

	Inclusion	Exclusion
Population	HCWs in healthcare settings	Individuals in non-healthcare settings
	Simulated participants in healthcare settings	
Concept	Involves an AI-driven system for automatic hand hygiene monitoring/recognition	Traditional methods only
		Non-AI electronic monitoring
		Purely conceptual “AI” mention
Context	Clinical settings	Limited to non-clinical lab settings without real-world validation
	High-fidelity simulated environments	
Types of source	Published: 2000-01-01 to 2025-09-30	Outside date range
	Peer-reviewed original research	Non-English language
	No geographical restrictions	Non-peer-reviewed
	No limitations on research design or quality	Non-original research
		Unavailable full text

Abbreviations: AI, artificial intelligence; HCWs, Healthcare workers.

#### 2.2.1 Population.

The population in this study is defined as HCWs (e.g., physicians, nurses, medical technicians) performing hand hygiene behaviors in healthcare institutions, as well as healthy volunteers or other individuals simulating the roles of HCWs.

#### 2.2.2 Concept.

The core concept of this review centers on AI technologies applied to the automated monitoring, recognition, or evaluation of the aforementioned hand hygiene behaviors. This encompasses, but is not limited to, systems utilizing computer vision, deep learning, sensor fusion, or related technologies.

#### 2.2.3 Context.

The review includes studies conducted in real or simulated healthcare environments, such as hospital wards (e.g., ICUs, general wards), operating rooms, emergency departments, or other acute and chronic care settings.

#### 2.2.4 Types of source.

This review includes English-language peer-reviewed original research articles published between January 1, 2000, and September 30, 2025. Letters to the editor, conference abstracts, editorials, commentaries, review articles, and other non-original publications are excluded. Studies for which full text cannot be accessed will also be excluded. No restrictions are applied regarding geographical region or study design.

### 2.3 Search strategy

The search strategy was developed in consultation with a librarian and the research team, adhering to the recommendations of the JBI [38]. Two experienced researchers (LXR and LY) conducted searches across the following databases: PubMed, Scopus, Embase, Web of Science, and IEEE Xplore. Search strategies were tailored for each database, combining keywords and subject headings related to AI-based hand hygiene compliance.

The core search strategy employed the following conceptual framework: (Artificial Intelligence OR Machine Learning OR Deep Learning OR Computer Vision OR Neural Network) AND (Hand Hygiene OR Hand Disinfection OR Hand Sanitization OR Handwashing). The comprehensive literature search was conducted on October 5, 2025, with complete search strategies for each database provided in S2 Appendix. To identify potentially omitted studies, backward citation tracking (snowballing) was performed on all included articles. Additional grey literature searching was completed via Google Scholar and ProQuest on October 15, 2025, with no subsequent updates to the search results. Grey literature searching was performed to improve search sensitivity and identify potentially eligible peer-reviewed studies missed by database searches; however, only studies meeting the predefined eligibility criteria were included.

### 2.4 Study selection

All retrieved records were imported into EndNote X9 for management and automatically deduplicated. Two independent reviewers (LXR and LY) subsequently screened titles, abstracts, and full texts against eligibility criteria, with documented reasons for exclusion. Inter-rater agreement was assessed using Cohen’s kappa coefficient (κ = 0.80). Discrepancies were resolved through discussion with a third reviewer (WPP).

Consistent with scoping review methodology [39], formal critical appraisal of included studies was not performed. This is because the primary purpose of this review was to map the breadth, characteristics, and research gaps of the available evidence in an emerging field, rather than to determine intervention effectiveness or exclude studies based on methodological quality. Nevertheless, to support interpretation, we extracted and summarized study design, setting, participant/sample characteristics, and validation context (e.g., technical validation, pilot clinical evaluation, or broader real-world implementation) where reported. The study selection process is summarized in the PRISMA-ScR flow diagram.

### 2.5 Data extraction

Data extraction was performed using a predefined standardized form in Microsoft Excel. Two investigators (LXR and LY) independently extracted the data, with any discrepancies resolved through team consensus. To ensure accuracy, two additional reviewers (WPP and CMH) conducted random verification checks of the extracted data. The final extracted dataset was reviewed and confirmed by all authors. Extracted data encompassed the following key elements: study characteristics (title, authors, publication year, country, study design), study context (healthcare setting type, participant types, and sample size), core features of AI technologies (technical approach, algorithmic architecture, monitoring functions), system performance metrics (e.g., accuracy, sensitivity), and reported challenges during implementation (technical, practical, and ethical). The complete extracted dataset from all included studies is available in S3 Appendix.

### 2.6 Data analysis

This study adopted the methodological framework proposed by Westphal et al. (2021) [40], integrating descriptive statistics and thematic analysis. All analytical steps were discussed and agreed upon by the research team. Specifically, descriptive statistics were first used to characterize the basic features of the included studies, including publication year, geographical distribution, study design, sample size range, and participant category. The results were presented as frequencies and percentages. Subsequently, thematic analysis was conducted following the framework outlined by Braun and Clarke [41], focusing on four core dimensions: technology types and characteristics, system performance metrics, implementation challenges, and future research directions and trends.

The findings from these analyses were synthesized and presented using tables and charts for clarity and accessibility. The detailed classification and coding framework used to synthesize the evidence is provided in S4 Appendix. In addition, to strengthen the technical synthesis and facilitate cross-study comparison, we developed two integrative visual summaries to represent the general framework and the comparative technical landscape of the included studies. These summaries are detailed in the Results section.

## 3 Results

### 3.1 Screening results

**Fig 1:**
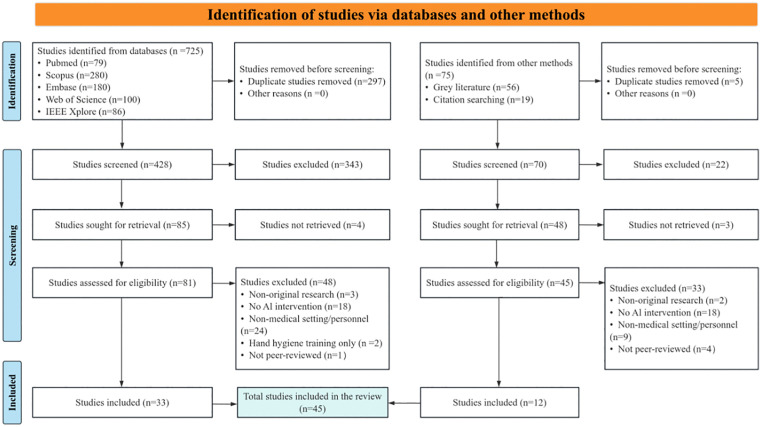
PRISMA 2020 flow diagram of study selection. Flow diagram showing the identification, screening, eligibility assessment, and inclusion of studies. A total of 800 records were identified, including 725 from databases and 75 from other methods. After removal of 302 records before screening, 498 records were screened, 126 full-text reports were assessed for eligibility, and 45 studies were included in the review. Abbreviations: AI, artificial intelligence; IEEE, Institute of Electrical and Electronics Engineers..

A total of 800 records were identified through database searching, grey literature searching, and citation tracking, including 725 records from databases and 75 records from other methods. Before screening, 302 records were removed, including 297 duplicate records identified from databases and 5 duplicate records identified from other methods. A total of 498 records underwent title and abstract screening, of which 365 were excluded. We sought to retrieve 133 reports for full-text assessment, and 7 reports could not be retrieved. Consequently, 126 full-text reports were assessed for eligibility, of which 81 were excluded. Ultimately, 45 studies [27,28,30,34,35,42–81] met the predefined inclusion criteria and were included in this scoping review. The specific reasons for exclusion at the full-text stage are provided in S5 Appendix. The detailed study selection process is illustrated in Fig 1.

### 3.2 Study characteristics

#### 3.2.1 Publication year and geographical distribution.

**Fig 2:**
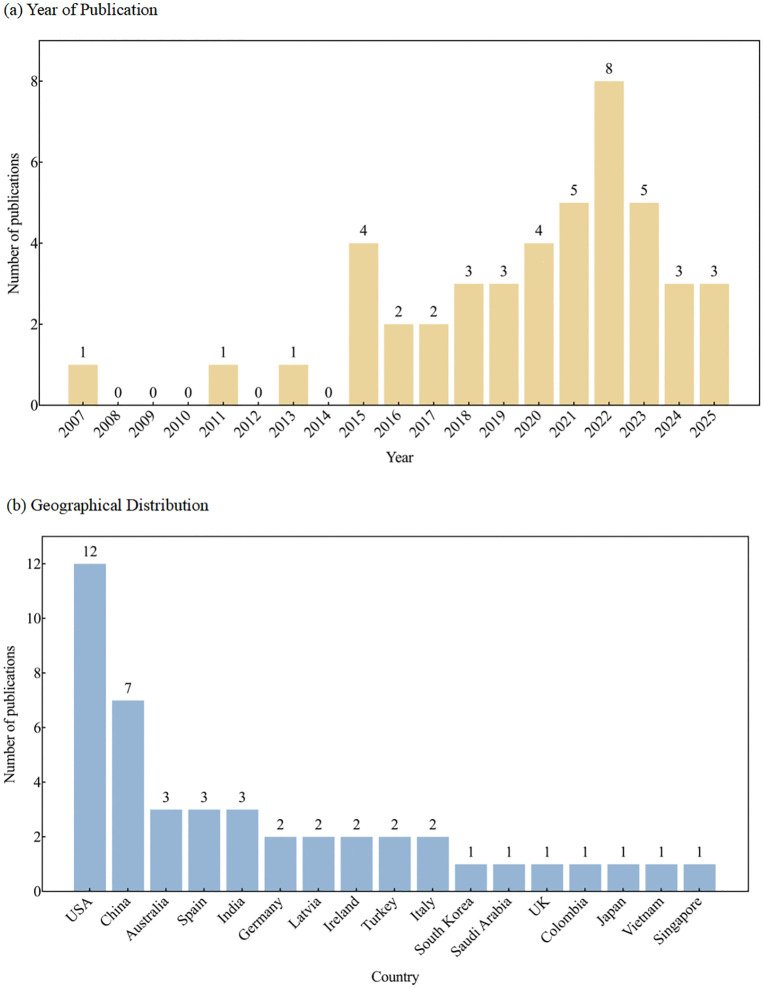
Publication year and geographical distribution. Figure (a) presents the distribution of included studies by year of publication. Figure (b) illustrates the geographical distribution of the study locations, with the bar heights representing the number of publications originating from each country. Abbreviations: USA, United States of America; UK, United Kingdom.

The included studies span publication years from 2007 to 2025, with 93.3% (42/45) published after 2015, reflecting a marked increase in research activity in this field over the past decade. Geographically, the majority of studies originated from the United States [30,34,49,59–61,63,65,70,77,80,81] (n = 12, 26.7%), followed by China [42,47,48,51,52,57,76] (n = 7, 15.6%). Australia [28,75,79], India [56,72,74], and Spain [58,66,67] each contributed three studies. Italy [27,62], Ireland [45,78], Turkey [50,73], Germany [53,64], and Latvia [54,68] each provided two studies. Single studies were contributed by South Korea [43], Saudi Arabia [44], the United Kingdom [35], Colombia [46], Japan [55], Vietnam [69], and Singapore [71]. These findings indicate a global distribution of research in this field, though contributions are exclusively from high- and middle-income countries, with no representation from low-income countries. Detailed distributions of publication years and geographical origins are presented in Fig 2.

#### 3.2.2 Study design and setting distribution.

Among the included studies, the distribution of study designs was as follows: experimental studies accounted for 37 studies (82.2%), quasi-experimental studies for 5 (11.1%), technical validation studies for 2 (4.4%), and diagnostic accuracy studies for 1 (2.2%). The healthcare settings covered included general wards (n = 35, 77.8%), ICUs (n = 13, 28.9%), emergency departments (n = 3, 6.7%), operating rooms (n = 2, 4.4%), dental clinics (n = 1, 2.2%), and general clinics (n = 1, 2.2%). Several studies encompassed multiple clinical environments, resulting in percentage totals exceeding 100%. High-risk infection settings, including ICUs, emergency departments, and operating rooms, were focal points of research attention. The distribution of study designs and settings is detailed in Table 2.

**Table 2:** Study design/validation categories and settings of included studies.

Characteristics	Studies, n (%)	References
**Research designs**		
Experimental studies	37 (82.2)	[27] [28,34,43,44,46–70,72,75,77–81]
Quasi-experimental studies	5 (11.1)	[35] [45,71,73,76]
Technical validation studies	2 (4.4)	[42] [74]
Diagnostic accuracy study	1 (2.2)	[30]
**Setting distribution**		
General wards	35 (77.8)	[34] [28,35,45–54,56–64,66–69,72–80]
Intensive care units	13 (28.9)	[42] [47,49,51,55,58,65,70,74–77,81]
Emergency departments	3 (6.7)	[30] [47,70]
Operating rooms	2 (4.4)	[27] [43]
Dental clinic	1 (2.2)	[44]
General clinic	1 (2.2)	[71]

#### 3.2.3 Study participants and sample characteristics.

Among the 45 included studies, at least 378 clinical HCWs, including physicians, nurses, and medical students, were explicitly reported as participants [42,45,47,65,68,73,76,77]. Additionally, some studies utilized healthy volunteers to simulate clinical HCWs [27,28,50,57–59,61,66,75], whereas other studies did not report the exact number of participants, reporting only overall sample sizes [30,34,35,43,44,46,48,49,51–56,60,62–64,67,69–72,74,78–81]. Reported participant numbers and sample characteristics varied substantially across studies. Video and image datasets also demonstrated considerable variation, spanning thousands to tens of thousands of frames across different studies [42,47,58,68,77]. Small-scale experimental investigations primarily focused on algorithm validation. Examples included 8 nurses generating 12,036 samples in an ICU setting [76], 22 HCWs recruited for cumulative observation in the Geneactiv Shadowing project [77], and 6 participants providing continuous 7-day data documenting 365 hand hygiene events via low-cost wearable sensors [58]. Moderate-scale clinical evaluations included a multi-camera system deployed across five departments capturing 165 video segments from 55 physicians and nurses [47]. Large-scale clinical validation was demonstrated in a burn center study monitoring 41 HCWs during 20,095 hand hygiene opportunities, documenting 15,202 compliance events [73]. The ICU-MH database contained 74,471 image frames from 20 HCWs [42]. In non-clinical technical development, one investigation utilized 32,471 publicly annotated video sequences for algorithm training [72], while another study conducted comparative experiments with 72 medical personnel at a medical school [68].

#### 3.2.4 Study maturity and validation context.

To improve interpretability, the included studies were also considered in terms of study maturity and validation context. Overall, the evidence base was dominated by early-stage investigations, with most studies focusing on technical development, controlled validation, or limited pilot testing rather than sustained real-world implementation. As shown in Table 2 and Table 3, experimental studies accounted for the majority of included articles, whereas only a small proportion were quasi-experimental, diagnostic accuracy, or technical validation studies. In addition, the validation context presented in Table 3 indicates that most studies were still at the stage of technical development, controlled validation, or limited pilot clinical evaluation. Only a few studies reflected broader real-world implementation. Sample sizes and evaluation conditions also varied substantially: several studies involved small numbers of participants or highly controlled datasets for algorithm development, while only a few studies reported larger-scale clinical evaluations or deployment in routine care settings. Accordingly, the reported performance of AI-based hand hygiene monitoring systems should be interpreted in light of study maturity, sample size, and validation context, and not assumed to reflect equivalent levels of clinical readiness or generalizability across studies.

**Table 3:** Technical pathways, characteristics, and validation context / study maturity of AI-based hand hygiene compliance monitoring.

References	Technology route	Core features	Validation context / Study maturity
[27]	Wearable sensors + Deep learning	Real-time motion capture, automatic evaluation of handwashing steps, quantitative compliance scoring	Technical development / laboratory validation
[30]	Computer vision + CNN	Real-time monitoring, dispenser-use recognition, compliance tracking	Pilot clinical evaluation
[42]	RGB video + 3D CNN	Continuous ICU surveillance, multi-person detection, automatic action recognition	Pilot clinical evaluation
[43]	2D/3D CNN + Optical flow fusion	Multi-person tracking, hand hygiene action classification, spatial cropping for accuracy	Technical development / laboratory validation
[44]	Pretrained CNN (Efficient Net) + Transfer learning	Step-level evaluation, AI–auditor agreement 90.9%, automatic feedback	Pilot clinical evaluation
[34]	Computer vision + depth sensing	HCW-patient contact detection, potential hand hygiene opportunity estimation, PPE compliance recognition	Technical development / laboratory validation
[28]	Privacy-preserving vision + Pose estimation	Skeleton-based motion tracking, anonymized data, accuracy analysis	Technical development / laboratory validation
[45]	Computer vision + Hand hygiene action training system	Automated hand hygiene assessment and feedback, video analysis	Pilot clinical evaluation
[35]	Video analytics + Real-time feedback	Automated sink-mounted system, WHO-step tracking, 193% quality improvement	Pilot clinical evaluation
[46]	MediaPipe landmarks + Logistic regression	99% accuracy, 3 ms inference, real-time duration tracking, low computational cost	Technical development / laboratory validation
[47]	Multicamera vision + skeletal tracking + machine learning	Handwashing gesture recognition, duration/frequency analysis, quality scoring, validation across hospital departments	Pilot clinical evaluation
[48]	Wearable sensors + Deep neural network fusion	Multi-level residual fusion, Spatio-temporal feature extraction, Real-time hand hygiene detection	Technical development / laboratory validation
[49]	Camera-based system + Deep learning + Sensor integration	Compliance monitoring, Automatic detection, Multi-sensor fusion, Hospital-grade accuracy	Technical development / laboratory validation
[50]	Spatio-temporal data + Deep learning models	Spatio-temporal data analysis, accurate hand hygiene procedure evaluation and classification	Technical development / laboratory validation
[51]	3D CNN + Probability smoothing + Video-based analysis	Step recognition, Real-time feedback, Automated assessment, Quality grading consistency	Technical development / laboratory validation
[52]	Self-attention CNN + Computer vision	Real-time hand hygiene detection, High spatial–temporal accuracy, Automatic recognition	Technical development / laboratory validation
[53]	Gas sensors + Machine learning	VOC pattern recognition, Handwash detection via odor profiles, Low-cost non-visual sensing	Technical development / laboratory validation
[54]	MobileNetV2 CNN + Two-stream + Recurrent GRU	RGB & optical flow fusion, Movement classification, Dataset generalization analysis	Technical development / laboratory validation
[55]	2D camera + Pose estimation + SVM classifier	Single-camera monitoring, Wrist motion tracking, Non-intrusive compliance detection	Technical development / laboratory validation
[56]	CNN (VGG16) + Transfer learning	Frame-based video analysis, WHO step detection, Real-time compliance feedback	Technical development / laboratory validation
[57]	YOLOv3-tiny + TensorRT + Jetson Nano	Gesture recognition for seven-step washing, Real-time detection, High accuracy (90%), Edge device deployment	Technical development / laboratory validation
[58]	Wrist-worn IMU sensors + Machine learning	Detect free and WHO-standard wash events, Low-cost wearable, Real-time motion analysis	Technical development / laboratory validation
[59]	Inertial sensors + Deep learning (CNN + LSTM)	Motion recognition, Temporal feature fusion, High detection accuracy	Technical development / laboratory validation
[60]	Depth sensors + CNN (InceptionV3) + NFMI tagging	Hand pose recognition, WHO-step compliance, Real-time feedback, Edge-device deployment	Technical development / laboratory validation
[61]	RGB camera + ResNet-18 CNN + AWS cloud deployment	Real-time hand pose classification, Low-cost cloud integration, Instant feedback to user	Technical development / laboratory validation
[62]	Depth camera + CNN (AlexNet-based) + Sliding window voting	Surgical gesture recognition, Real-time compliance evaluation, Depth-based robustness, Training feedback for surgeons	Pilot clinical evaluation
[63]	Multi-sensor network + Microcontroller + Wireless communication	Automatic hygiene data collection, Real-time monitoring, Low-power IoT deployment	Technical development / laboratory validation
[64]	Interactive hand segmentation + CNN (U-Net)	Accurate hand-region detection, Robust segmentation under occlusion, Vision-based compliance tracking	Technical development / laboratory validation
[65]	Statistical analysis + Random forest + Logistic regression	Compliance feature prediction, Risk factor identification, Data-driven behavioral modeling	Technical development / laboratory validation
[66]	RGB camera + Motion & skin segmentation + SVM classification	Gesture recognition, Quality scoring, Real-time visual feedback, Multi-class SVM accuracy (97%)	Technical development / laboratory validation
[67]	RGB video + Feature extraction + Multi-class SVM ensemble	Multi-class gesture classification, Quality scoring, Automated evaluation, Accuracy >95%	Technical development / laboratory validation
[68]	Smartphone camera + Neural network (MobileNetV2) + Real-time feedback	Visual/audio feedback guidance, Compliance improvement, Frame-level motion recognition, Real-time quality assessment	Technical development / laboratory validation
[69]	RGB cameras (6 views) + Self-supervised learning (AmDim + ResNet)	Multi-view gesture recognition, Fine-grained motion analysis, Cross-view generalization, High Macro F1 performance	Technical development / laboratory validation
[70]	Depth sensors + CNN (ResNet-152) + Spatial Transformer Network	Non-intrusive vision tracking, Hand hygiene classification, Real-time compliance analytics, 95% accuracy	Technical development / laboratory validation
[71]	RGB cameras + Deep learning (CNN + Object tracking)	Real-time compliance detection, Staff movement tracking, Automated feedback, Workflow optimization	Pilot clinical evaluation
[72]	RGB camera + CNN (ResNet50) + Deep learning	Gesture recognition, Step detection, Real-time feedback, Visual instruction for compliance	Technical development / laboratory validation
[73]	Wearable badges + Motion sensors + Cameras + Deep learning	Automated event tracking, Real-time reminders, Compliance analytics, Continuous performance improvement	Broader real-world implementation
[74]	LoRaWAN IoT + RFID sensors + Cloud dashboard	Long-range wireless tracking, Data visualization, Real-time event logging, Low-power architecture	Pilot clinical evaluation
[75]	mmWave radar + Deep learning (BiLSTM + CTC loss)	Device-free RF sensing, Nine-step gesture tracking, Weakly supervised learning, Privacy-preserving sensing	Technical development / laboratory validation
[76]	CCTV cameras + Hand landmark detection (MediaPipe) + SVM classifier	Multi-view video recognition, Step classification, Real-time scoring and alerting, Integration with hospital IoT	Pilot clinical evaluation
[77]	Wearable wrist sensors + machine learning	Hand hygiene event detection, duration estimation, technique recognition, hospital-floor observation	Pilot clinical evaluation
[78]	RGB-D camera + CNN + Skeleton tracking	Posture recognition, Depth-assisted segmentation, Real-time motion analysis	Technical development / laboratory validation
[79]	Depth sensor + Decision tree + Background subtraction	Contactless monitoring, Gesture classification, Real-time feedback, Lightweight design	Technical development / laboratory validation
[80]	Smartwatch IMU sensors + Bluetooth beacons + Machine learning	Gesture detection, Real-time reminders, Compliance tracking, Data synchronization	Technical development / laboratory validation
[81]	Accelerometer + Gyroscope + Machine learning (k-NN classifier)	Technique classification, Duration estimation, Dual-wrist sensing, 93% accuracy	Technical development / laboratory validation

Abbreviations: AI, artificial intelligence; CNN, convolutional neural network; 3D CNN, three-dimensional convolutional neural network; RGB, red-green-blue; RGB-D, red-green-blue plus depth; ICU, intensive care unit; HCW, healthcare worker; PPE, personal protective equipment; WHO, World Health Organization; VOC, volatile organic compounds; GRU, gated recurrent unit; YOLO, you only look once; TensorRT, tensor runtime; IMU, inertial measurement unit; LSTM, long short-term memory; NFMI, near-field magnetic induction; AWS, Amazon Web Services; IoT, Internet of Things; U-Net, U-shaped network; SVM, support vector machine; mmWave, millimeter wave; BiLSTM, bidirectional long short-term memory; CTC, connectionist temporal classification; CCTV, closed-circuit television; LoRaWAN, long range wide area network; RFID, radio-frequency identification; k-NN, k-nearest neighbors.

In addition, important heterogeneity was observed in how study outcomes, reference standards, and validation strategies were defined across the included literature. Some studies evaluated hand hygiene event detection or opportunity recognition, whereas others focused on procedural step recognition, duration compliance, technique quality, or post-implementation compliance improvement. Reference standards also varied, including direct human observation, expert video annotation, sensor-derived event logs, and internally constructed technical benchmarks. Validation strategies ranged from laboratory-based algorithm development and internal dataset testing to pilot clinical evaluations and limited real-world implementation. This heterogeneity further limits direct comparison of reported performance metrics across studies and reinforces the need to interpret accuracy estimates in light of study maturity, evaluation target, and validation context.

To facilitate interpretation of the reported performance measures, Table 4 summarizes the included studies according to validation context and evidence maturity, distinguishing technical development/laboratory validation, pilot clinical evaluation, and broader real-world implementation. This framework provides additional context for understanding why reported accuracy and related metrics should not be directly compared across studies or interpreted as equivalent indicators of clinical readiness.

**Table 4:** Framework for interpreting reported performance metrics according to validation context and evidence maturity.

Evaluation type / validation context	Typical study characteristics	Common performance measures reported	Interpretation caveat
Technical development / laboratory validation	Prototype-stage systems evaluated under controlled conditions, often with limited participant diversity, constrained scenarios, and internally defined reference standards	Accuracy, sensitivity, specificity, precision, recall, F1 score, processing or inference speed	These metrics mainly reflect technical performance under constrained conditions and should not be interpreted as evidence of clinical reliability, broad generalizability, or implementation readiness
Pilot clinical evaluation	Small-scale deployment in a single ward, unit, or institution, with limited workflow exposure and short observation periods	Detection accuracy, agreement with human observers, compliance-related measures, feasibility or usability observations	Findings may suggest early implementation potential, but they remain insufficient to establish robust effectiveness, scalability, or sustained integration into routine clinical practice
Broader real-world implementation	Use in routine care environments with broader operational exposure, repeated use over time, and greater interaction with real-world workflow conditions	Compliance trends, operational feasibility, real-world monitoring performance, workflow-related outcomes	These studies provide more practice-relevant evidence, but heterogeneity in outcome definitions, implementation conditions, and comparator standards still limits direct cross-study comparison

Note: F1 score = F1 score (harmonic mean of precision and recall).

### 3.3 AI technical pathways and characteristics

**Fig 3:**
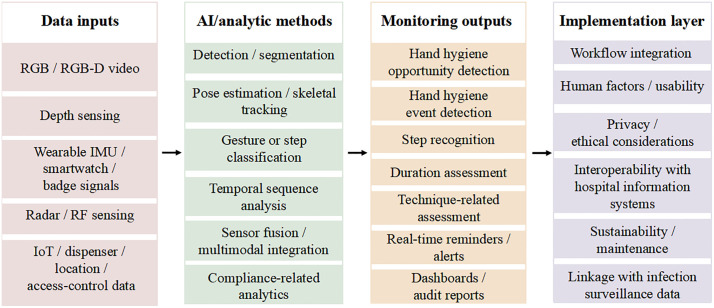
General framework of AI-based hand hygiene monitoring systems in healthcare settings. General framework of AI-based hand hygiene monitoring systems in healthcare settings, showing data inputs, analytic methods, monitoring outputs, and implementation considerations.Abbreviations: AI, artificial intelligence; RGB, red-green-blue; RGB-D, red-green-blue plus depth; IMU, inertial measurement unit; RF, radio frequency; IoT, Internet of Things.

**Fig 4:**
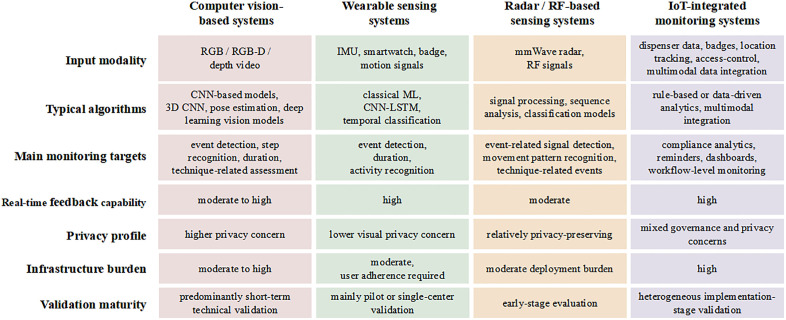
Comparative technical landscape of AI hand hygiene monitoring approaches represented in the reviewed studies. Comparison of the main AI-based hand hygiene monitoring approaches reported in the reviewed studies, including computer vision-based, wearable sensing, radar/radio frequency-based, and IoT-integrated systems. Abbreviations: AI, artificial intelligence; RGB, red-green-blue; RGB-D, red-green-blue plus depth; CNN, convolutional neural network; 3D CNN, three-dimensional convolutional neural network; IMU, inertial measurement unit; ML, machine learning; LSTM, long short-term memory; RF, radio frequency; mmWave, millimeter wave; IoT, Internet of Things.f Things.

To clearly delineate the technical applications and features of AI in hand hygiene compliance monitoring, we extracted the technical pathways and core characteristics from each included study, as summarized in Table 3. To complement the study-level summaries, Fig 3 presents a general framework of AI-based hand hygiene monitoring systems, illustrating the relationship between data inputs, analytical methods, monitoring outputs, and implementation layers. Fig 4 provides a comparative overview of the major technical routes represented in the included studies, highlighting differences in input modality, algorithmic strategies, monitoring targets, feedback capability, privacy profile, infrastructure burden, and validation maturity.

#### 3.3.1 Computer vision-based systems (24 studies, 53.3%).

As the predominant technical approach, these systems utilize cameras for non-contact monitoring, offering the key advantage of objectively recording hand hygiene compliance, movement accuracy, and duration. Functionally, such systems comprehensively cover hand hygiene event detection (24 studies), technique classification (17 studies), duration estimation (15 studies), and real-time feedback (10 studies), thereby providing multi-dimensional data for healthcare-associated infection surveillance.

System performance is closely linked to potential clinical applicability, particularly in studies approaching clinical application: personalized models trained on specific HCWs can achieve high recognition accuracy, such as 95% in ICU settings [76], although this level of performance was reported under setting-specific conditions and does not imply comparable generalizability across users or institutions. In contrast, generalizable models designed for cross-user application exhibit reduced performance due to variations in hand size and washing habits, with accuracy potentially declining to 56% [76]. This finding underscores the importance of model adaptability for future real-world deployment rather than indicating equivalent readiness across all study contexts.

Moreover, privacy concerns represent a significant challenge in clinical implementation. Multi-camera fusion [47] may help reduce occlusion in complex clinical environments, with reported recognition gains of 12%–18%.

#### 3.3.2 Wearable sensor-based systems (11 studies, 24.4%).

These systems are suitable for mobile clinical settings with limited camera coverage, such as ICUs and general wards. Their advantages include portability and extended battery life, with low-power designs enabling continuous operation for 7–14 days [58]. The primary functions of these systems focus on estimating hand hygiene duration (11 studies) and detecting the occurrence of the action (8 studies).

A key limitation for clinical application is their relatively low specificity, which renders them susceptible to false alarms triggered by similar hand movements, such as writing or operating medical instruments. This results in a specificity that is 5%–10% lower than that of computer vision-based systems [77]. To improve monitoring accuracy, studies have demonstrated that dual-wrist configurations [77] capture bimanual coordination more effectively than single-wrist setups, increasing recognition accuracy by 8%–15%. Furthermore, some systems, when integrated with GPS positioning, have achieved linked monitoring of “hand hygiene location and behavior,” reporting false alarm rates below 5% [80]. This capability provides valuable managerial insights for analyzing the relationship between compliance rates and specific clinical locations.

#### 3.3.3 Radar/radio frequency-based systems (4 studies, 8.9%).

This category of technology provides a privacy-centric alternative for non-contact monitoring, as its operational principle does not involve capturing visual data. This attribute makes it particularly suitable for deployment in privacy-sensitive environments such as patient rooms. For instance, one implementation employs gas sensors [53] to support the verification of hand hygiene events by rapidly measuring the volatile alcohol concentration emitted from hand rub solutions, achieving a response time of less than one second.

Nevertheless, the technological maturity of these systems requires further development. Their performance stability is vulnerable to interference in clinical settings; specifically, recognition accuracy can drop to a range of 68% to 75% [75] when multiple individuals are present simultaneously. This limitation currently hinders their broader adoption in large-scale applications.

#### 3.3.4 IoT-integrated AI systems (6 studies, 13.3%).

This category represents the most comprehensive management solution, establishing a closed-loop system that spans from monitoring to intervention by integrating intelligent hand sanitizer dispensers with multi-sensor networks and communication technologies such as Long Range Wide Area Network (LoRaWAN). These systems not only automate the generation of compliance rate reports for HCWs [74] but also employ both mandatory and guided measures, including access control linkages. Some integrated approaches have reported compliance improvements of 30% to 45% [49], suggesting possible behavioral benefits under specific implementation conditions, although broader real-world validation remains limited.

In a burn center study, deployment of such a system combined with weekly individualized feedback increased compliance from 58.5% to 80.5% [73], suggesting that implementation strategies may enhance the impact of these systems under specific deployment conditions. However, deployment costs may still represent an important barrier to widespread hospital-scale implementation.

### 3.4 Cross-cutting implementation dimensions

To move beyond technological classification alone, we additionally synthesized cross-cutting implementation dimensions across the included studies, including workflow integration mode, feedback mechanisms, human factors, organizational readiness, fairness-related considerations, and key implementation concerns (Table 5).

**Table 5:** Cross-cutting implementation dimensions of AI-based hand hygiene monitoring systems reported in included studies.

References	Workflow integration mode	Primary users / affected staff	Feedback mechanism	Human factors considerations reported	Organizational readiness / implementation requirements	Fairness / differential performance considerations	Key implementation concerns
[27]	Real-time prompting / training	Surgeons, trainees, HCWs performing surgical handwashing	Real-time gesture-level feedback; overall score	User-centered GUI was developed after observing staff habits & discussing interface preferences with medical staff; designed to facilitate immediate correction during training	depth camera mounted above washbasin, embedded Jetson Nano platform, fixed positioning & controlled capture zone	No subgroup fairness analysis; dataset included male & female volunteers of different heights, but performance stratification across user groups was not reported	Setup sensitivity, limited dataset, likely dependence on controlled positioning & predefined gesture sequence
[30]	Passive monitoring	HCWs entering/exiting rooms	No direct feedback; system used for automated observation & comparison with human auditing	Privacy-focused depth sensing; no identifiable video; less workflow disruption than tags	16 ceiling-mounted depth sensors across a hospital unit, continuous image capture, model training on large annotated image set	No subgroup fairness analysis reported; no analysis across staff roles or demographic groups	Installation burden, large annotated dataset, dispenser use only (not full technique)
[42]	Retrospective camera assessment; future IoT scoring/alerts	Clinicians in ICU	No real-time feedback; study discusses future scoring & alert system	Human factors not systematically assessed; side-view CCTV led to hand detection failure from angle, foam occlusion, & motion blur	ICU CCTV integration, multiview setup, frame-by-frame annotation, & setting-specific calibration; for eventual hospital IoT integration	Strong differential performance issue explicitly reported: personalized models performed much better than generalized models; study concludes recognition is subject-dependent & cross-subject adaptation is needed	Poor generalizability, camera-angle dependence, occlusion, class imbalance & ICU integration challenges
[43]	Passive OR video surveillance with retrospective detection	Anesthesia personnel in operating rooms	No direct user feedback; positioned as monitoring support for compliance review	Human factors not directly assessed; paper notes behavior may change during observation periods & OR context is complex	OR video capture over months, 2D person detection plus 3D CNN pipeline, handling of multi-person scenes, upper-body cropping & spatial extent fixing; evaluated in a single OR with additional simulated data	No fairness analysis; performance varied with scene complexity, overlapping staff, identical scrubs, & occlusion by equipment	Single-site validation, occlusion, person misalignment, complex multi-person scenes, limited generalizability & no direct moment-based monitoring
[44]	Retrospective video step assessment	Dental students & dental assistants; infection control auditors as comparison assessors	No real-time feedback in current implementation; retrospective AI evaluation compared with auditors; future real-time feedback proposed	Privacy, data confidentiality, ownership, & acceptance were explicitly discussed; only hand movements were recorded to maintain anonymity	wall-mounted camera, labeled local dataset, auditor comparison workflow, & likely retraining for other clinics; single-clinic deployment	No fairness analysis; study notes single-location training limits generalizability to other clinics & environments	Single-site design, local labeling dependence, privacy/confidentiality concerns & possible divergence from real-time practice
[34]	Passive background surveillance for bedside contact & PPE compliance monitoring	HCWs entering rooms & interacting with patients	Automated detection; no direct feedback	Strong privacy-focused design emphasized; no recognizable images retained; no direct evaluation of staff acceptance	multiple Kinect cameras per room, fiducial markers, real-time tracking software, & room-specific installation/calibration; designed mainly for ICU-like environments	No subgroup fairness analysis; system could not distinguish HCWs from visitors without additional identifiers; performance may vary with lighting & skin-tone-like glove colors	Multi-camera burden, occlusion, limited field of view, visitor/staff ambiguity & ICU-specific design
[28]	Simulated bedside automated observation	Healthy volunteers simulating HCWs & patients	No implemented bedside operational feedback in the study; study notes potential future real-time feedback & reporting	Privacy protection is central: depth imagery used, patient face excluded, no RGB storage needed for deployment; study notes surveillance in clinical areas would require great care & sensitivity	Single Kinect above bed; controlled laboratory setup; would require careful deployment in real clinical environments; model performance may be affected by gloves, multiple workers, & real-world complexity	No subgroup fairness analysis reported; potential performance variation with glove color, multiple workers, & uncontrolled real clinical settings acknowledged	Simulation-only validation, limited real-world complexity, glove/skin segmentation dependence, privacy concerns & uncertainty with multiple workers
[45]	Real-time ward-based training	Ward-based HCWs; participation voluntary & anonymous	Real-time on-screen feedback using pass/fail result & pose-level indicators	Hands-only capture preserved anonymity; feedback increased participation; reviewer fatigue noted	ward-based device placement, live camera/screen unit, voluntary staff engagement, & ongoing deployment in clinical areas; larger studies needed	No fairness analysis; because participation was voluntary & anonymous, differences across staff subgroups could not be assessed	Small pilot, self-selection bias, no subgroup analysis, limited sample & need for sustained engagement
[35]	Hybrid monitoring & feedback at sink locations, combining continuous auditing with real-time & team-level feedback	HCWs using sinks in an active surgical unit	Real-time sink feedback; weekly team reports	Privacy-protective design noted: camera faced downward & no identifying information visible; some staff engaged positively while others ignored the system; peer-to-peer learning & reflection reported	installation of AVA units over all ward sinks, prolonged operation across phases, integration into local auditing routines, & maintenance of feedback strategy	No subgroup fairness analysis; Anonymous design prevented subgroup attribution; individual vs team effects unclear	Installation & maintenance burden, inability to measure ABHR opportunities, behavior returned to baseline after feedback removal, possible cognitive offloading, variable engagement
[46]	Real-time technique monitoring & feedback during handwashing/rubbing procedure	HCWs voluntarily participating in real-time testing	Real-time interface with step recognition, timing display, & red/green immediate feedback	Participants found the system somewhat non-intuitive; difficulties included uncertainty about when to start/finish, left-handed sequencing differences, moving out of view, & hand orientation issues; privacy preserved by recording hands only with consent	correct camera positioning, unobstructed hand view, horizontal alignment relative to sink, adequate lighting & processing capability; scalable if infrastructure conditions are met	Authors explicitly aimed to reduce bias from camera distance & hand size via centroid normalization; however, no formal subgroup fairness analysis was reported; left-handed users & alternate hand orientations affected performance	Sensitivity to camera angle/lighting, non-standard hand orientations, step confusion, intuitiveness issues & setup dependence
[47]	Multicamera sink monitoring	Physicians & nurses performing handwashing in hospital departments	Automated quality scoring vs expert annotations; no direct alerts	Human factors not systematically assessed; designed to emulate infection-control specialist scoring rather than user experience	three synchronized cameras around each basin, skeletal tracking, gesture-category model, smoothing/counting algorithms, & deployment across hospital departments	No subgroup fairness analysis; dataset included both male & female staff, but performance was not stratified by role, sex, or department	Multicamera installation burden, likely dependence on fixed positioning, controlled basin-centered capture, & untested broader real-world scalability
[48]	Retrospective handwashing action recognition	General users/participants in custom & public datasets; not clearly real healthcare deployment	No real-time feedback; framed primarily as automated recognition/quality assurance	Human factors not directly assessed; study notes data scarcity, variability across individuals & contexts, & need for efficient models	video datasets, model training pipeline, & computing resources; focused on algorithm development rather than clinical deployment workflow	Explicit concern about limited data diversity & difficulty generalizing across different individuals & contexts; no subgroup fairness analysis	Limited real-world validation, dataset scarcity, unclear healthcare workflow integration, & uncertain generalizability across environments/users
[49]	Hybrid monitoring & enforcement across sinks, dispensers, & beds using cameras, sensors, & wearables	Individual hospital staff wearing wrist/bicep module; teams/departments can also be monitored	Real-time pass/fail alerts via vibration & LED to staff module; central server records compliance data for dashboards/management use	Directly surveillance-oriented; privacy concerns of video systems are discussed in literature review, but user acceptance, alert burden, & perceived intrusiveness are not evaluated in the study	RFID-tagged wearable staff module, sink/rub/bed modules, WiFi central server, camera microcontrollers, IR/water/force sensors, & hospital-wide infrastructure	No subgroup fairness analysis; the study cites prior skin-color bias limitations in earlier vision work, but do not test differential performance across staff groups in CareHAI itself	High infrastructure complexity, wearable dependence, real-time enforcement burden, privacy/surveillance implications, & no demonstrated real-world hospital implementation outcomes
[50]	Real-time or near-real-time sink-based procedure evaluation using vision-based frame-level & spatio-temporal models	HCWs; dataset collected from 2 participants in 2 locations	Automated 8-gesture classification; no direct alerts	Camera placement & usability are explicitly considered; overhead placement chosen to reduce occlusion & water-contact risk; no user acceptance evaluation	Kinect 2.0 RGB-D camera mounted above sink, real-time multithreaded acquisition, background subtraction/ROI extraction, & consistent spatial setup across sites	Very limited participant diversity (2 participants only); no formal subgroup fairness analysis; performance may vary with camera position, occlusion, & non-standard gestures/durations despite the study’s robustness claims	Extremely limited sample diversity, dependence on ideal overhead setup, small participant pool, & uncertain generalizability to broader clinical practice
[51]	Retrospective/nearline video-based quality assessment in hospitals & communities, with potential practical monitoring use	HCWs in hospitals & civilian participants in community settings	No direct real-time feedback implemented; system outputs step recognition, duration assessment, & quality grading	Human factors not systematically studied; study intentionally normalized background/clothing variation & excluded blurred/out-of-view videos, which suggests controlled input requirements rather than real-use burden evaluation	cellphone video capture, expert-labelled training data, 3D CNN-based assessment scheme, & setting-specific validation across hospital/community scenes	One of the strongest differential-performance examples: performance & agreement were better in hospital professionals than in community users, indicating context- & population-sensitive variation; no formal fairness analysis reported	Performance drop outside controlled hospital setting, reliance on cleaner views & trained users, exclusion of low-quality videos, & weaker external agreement in community use
[52]	Retrospective video-based handwashing action recognition with potential future monitoring use	General users/participants in benchmark video datasets; no real HCWs deployment reported	No real-time user-facing feedback implemented; positioned as automated action recognition for monitoring	Human factors not evaluated; emphasis is on algorithm performance rather than usability, acceptability, or workflow fit	curated benchmark video datasets, frame extraction, & substantial model training; primarily an algorithm-development study rather than a deployment study	No subgroup fairness analysis; only 3 participants contributed to the study’s own dataset, & generalizability across users/settings was not systematically assessed	Dataset-limited development, unclear real-world deployment pathway, small contributor pool in own dataset, uncertain external generalizability
[53]	Sensor-based monitoring near dispenser stations, with comparison between stationary & wearable setups	Participants simulating HCWs; portable tag worn on surgical gown in pilot project	No implemented real-time behavioral feedback in the evaluated system, but portable tag includes blinking LED for event registration & study discusses possible future reminders/alerts	Anonymous monitoring is explicitly emphasized as an advantage over video surveillance; wearable option may reduce privacy concerns but introduces behavior-dependent signal variability; user acceptance not formally studied	commercial gas sensors, stationary sensor array or portable tag, microcontrollers, WiFi/database/file-server infrastructure, & time-series model training; stationary setup performed better than portable setup	No subgroup fairness analysis; study note that portable-tag performance is influenced by wearer behavior/location after disinfection, indicating person-dependent variability	Infrastructure burden, portable setup instability, pilot/simulated setting rather than routine clinical deployment, classification difficulty for correct vs incorrect technique
[54]	Retrospective movement classification across datasets	Medical students in lab dataset; hospital staff in real-life dataset; multiple user groups across Kaggle/METC/hospital datasets	No real-time feedback in this paper; study focuses on movement classification performance across datasets	Human factors indirectly highlighted through real-world complexity: imperfect execution, variable lighting, occlusion, & incomplete performance are common in hospital data; no formal acceptability/usability study	dataset annotation, train/test split by unseen users & unseen locations, & smartphone-feasible CNN architectures; demonstrates strong dependence on dataset quality & context	Strongest fairness/generalizability signal in this batch: model performance drops sharply on more complex & hospital datasets, & fails to generalize to unseen real-life videos; performance varies across users & locations	Poor real-world generalizability, dataset dependence, context sensitivity, & high risk of overstating performance from lab-based datasets
[55]	Single-camera ICU hallway surveillance	Medical staff moving through ICU hallways; multiple persons tracked simultaneously	No direct bedside/user feedback; designed for observational approximation of hand disinfection events rather than intervention	Non-intrusive design is a central motivation; avoids requiring staff to wear devices & aims to reduce behavior change from observation, but acknowledges detection challenges from occlusion, orientation, & partial visibility	single ceiling-mounted camera, SSD, Jetson Nano, WiFi connectivity, pose estimation, tracking, & manual annotation; chosen specifically to minimize sensor clutter in real ICU environment	No subgroup fairness analysis; performance is likely affected by body orientation, occlusion, distance from camera, & multiple disinfection sources; only approximate compliance estimation is intended	Occlusion, inconsistent tracking IDs, limited fine-detail capture, approximation rather than precise event recognition, & uncertain performance across users/positions
[56]	Real-time sink-top technique monitoring	Users performing WHO handwashing steps in top-view basin videos; not clearly real clinical deployment	Real-time procedural feedback is explicitly intended: model checks whether WHO steps are completed correctly & can notify the user if handwashing is incorrect	Authors explicitly aim to reduce sensitivity to lighting & skin color by converting frames to grayscale; however, user intuitiveness, acceptability, & workflow integration are not evaluated	top-angle video capture, frame extraction, preprocessing to grayscale, modified VGG16 transfer-learning pipeline, & controlled basin-centered imaging conditions	No subgroup fairness analysis; grayscale preprocessing is intended to reduce variation from skin color/brightness, but broader fairness across users/settings is not assessed	Controlled dataset dependence, likely sensitivity to camera viewpoint/setup, limited real-world validation, & unclear scalability beyond scripted sink-based videos
[57]	Real-time sink-side/video-based monitoring & step recognition	Medical staff / hospital users performing seven-step handwashing	Real-time visual feedback on whether gestures are standard during washing; small display + camera terminal	Human factors not systematically assessed; study notes future need for a better user interface, feedback on correctness & duration, & voice prompts	camera, Jetson Nano edge device, TensorRT acceleration, packaged executable deployment, & fixed monitoring terminal with display	No subgroup fairness analysis; dataset included people of different ages, body types, & genders, but no stratified performance reporting was provided	Controlled dataset dependence, need for optimized UI/voice prompting, fixed terminal setup, unclear generalizability to routine clinical use
[58]	Wearable activity monitoring for handwashing moments	General users wearing commercial smartwatches/smartbands; not restricted to a single clinical role	No implemented real-time bedside feedback in the study itself; designed to support future personalized recommendations & reminders	Privacy is explicitly emphasized because only accelerometer & gyroscope data are used & microphones are avoided; wearable use is described as user-transparent	continuous smartwatch-based inertial sensing, ad hoc capture app/server infrastructure, labelled free-living data, & sufficient user-specific training data for best performance	Strong differential performance issue reported: user-dependent models were effective for both WHO & free washes, whereas user-independent models were much weaker for free-wash detection	Limited user-independent generalizability, especially for free washing; dependence on larger labelled datasets; ongoing need for personalized modelling
[59]	Wearable monitoring; potential just-in-time intervention	Wearers of wrist devices in healthcare, food business, or daily-life settings	No deployed reminder system in the study, but the study frames the system as enabling just-in-time alerts, accountability, & personalized feedback	Wearables are presented as location-flexible & less constrained than in-situ cameras/sensors; user acceptability not evaluated	smartwatch/fitness tracker inertial sensing, neural-network model, representative set construction, & robustness testing against unseen NULL activities	Explicitly addresses person/context generalization through out-of-distribution handling for unseen activities; no formal subgroup fairness analysis across demographic/staff groups reported	Risk of false positives from unseen NULL activities, dependence on representative training data, & no direct evaluation of real-world alert/feedback effects
[60]	Hybrid monitoring & enforcement combining “when” (zone/dispenser entry-exit) & “how” (WHO-step quality)	Tagged individual staff members; hospital management also receives compliance outputs	Immediate red-green LED/voice alerts; central dashboards	Surveillance/enforcement orientation is strong; privacy concerns of video systems are discussed in framing, but user acceptance, alert burden, & perceived intrusiveness are not evaluated	NFMI readers/tags, depth camera, edge device such as Raspberry Pi/Jetson, server/cloud integration, & substantial hardware/infrastructure deployment	No subgroup fairness analysis; study notes future need to gather staff/patient feedback & evaluate usability/acceptability in real hospitals	High infrastructure complexity, real-time enforcement burden, incomplete real-hospital validation, & unresolved usability/acceptability questions
[61]	Real-time vision-based procedure evaluation	Handwashing subjects/users performing WHO poses; not clearly actual frontline clinical deployment	Live monitor inference; only high-confidence poses shown	Human factors not systematically studied; system was intended to provide instantaneous correction, but background simplification & controlled capture indicate limited evaluation of real-use burden	AWS SageMaker/S3 training workflow, AWS DeepLens camera, Lambda integration, monitor display, & sufficiently diverse image data for retraining	Differential performance across users/conditions is explicitly shown: Model 1 degraded on new subjects & different lighting, & performance improved only after adding more diverse training data; no formal fairness analysis reported	Controlled-background dependence, sensitivity to subject/environment variation, need for more diverse data, & uncertain performance in real sinks/bathroom settings
[62]	Real-time surgical sink-side assessment	Surgeons or surgical staff performing hand cleaning	System designed to control/assess how carefully hands are cleaned; no detailed bedside alerting workflow reported in the snippet	Human factors not systematically assessed; framed around improving carefulness of cleaning rather than user experience	Likely fixed vision-based sink setup for surgical hand-cleaning assessment; conference-paper setting suggests prototype rather than mature deployment	No subgroup fairness analysis	Early-stage conference prototype, limited implementation detail, unclear real-world workflow integration & user acceptability
[63]	Real-time sink-side handwashing monitoring	HCWs / hospital staff	Automated verification of handwashing procedure against WHO motions; intended to encourage compliance, but no mature feedback architecture described	No formal human-factors evaluation reported; system premise explicitly relies on observation increasing compliance	Jetson TX2, embedded camera, image-processing pipeline, controlled capture conditions, background handling, & trained SVM models	No subgroup fairness analysis; data collected in well-lit, white-background, close-range setting, so broader generalizability across users/settings is uncertain	Strong dependence on controlled background/lighting, manual preprocessing pipeline, prototype-stage implementation, & unclear performance in busy real clinical environments
[64]	Dispenser-area monitoring with gesture recognition	Users performing hand disinfection near a dispenser; 21 subjects in evaluation	Real-time feedback at 30 fps is explicitly reported	Human factors not directly assessed; system is positioned as an optical assistance tool to reduce labor-intensive direct observation & Hawthorne-effect limitations	Kinect v2 depth/RGB sensing, downward-facing mounted sensor above dispenser area, colored-glove-assisted training-label generation, two-stage deep-learning pipeline, & controlled lighting aids/reflectors	No subgroup fairness analysis; evaluated with 21 subjects, but no stratified analysis by staff role or other user subgroups	Dependence on sensor placement & lighting control, training-labeling setup using colored gloves, occlusion/interaction complexity, & lack of broader implementation evaluation
[65]	Predictive/retrospective compliance analytics using location-system & dispenser-activation data	Tagged care providers in a 30-bed ICU; infection-control teams/managers are likely indirect users of predictions	No direct user feedback in the reported study; study propose future just-in-time alerts, targeted auditing, & training recommendations	Human factors & staff acceptance not evaluated; surveillance/prediction implications are present but not discussed empirically	BLE badges, room-level indoor positioning, smart dispenser activation logs, two months of ICU data, & machine-learning analytics pipeline	Performance differences were explored across contextual features (entry/exit behavior, room location, recent history), but no fairness analysis across demographic or staff subgroups was reported	Predictive rather than directly observational system, dependent on badge/dispenser infrastructure, possible surveillance concerns, & no evaluation of how alerts affect staff behavior or culture
[66]	Sink-based quality assessment	Handwashing users in healthcare or food-preparation settings	High-level analysis measures time spent in each pose to assess washing quality; no explicit user-alert interface described in the snippet	Robustness to lighting, occlusion, reflections, & sink-surface variation is a central design concern; no formal usability/acceptability study reported	camera-based sink observation, combined skin+motion segmentation, particle-filter tracking, optical-flow/motion descriptors, & SVM ensemble classification	No subgroup fairness analysis; system explicitly addresses variability in lighting & sink materials, but not user subgroup performance	Sensitivity to real-world reflections & occlusion, technical complexity of segmentation/tracking pipeline, & unclear large-scale deployment readiness
[67]	Sink-based quality assessment	Handwashing users in healthcare environments; not clearly tied to a specific staff subgroup	No explicit user-facing alerting interface described; system monitors hand motions to assess whether the six-step technique is followed	Human factors not systematically assessed; method explicitly addresses difficult real-world sink conditions such as lighting variation & reflections from stainless-steel surfaces	fixed camera over sink, robust hand/arm segmentation using combined skin & motion analysis, ROI extraction, & SVM-based pose classification; likely sensitive to deployment setup & lighting calibration	No subgroup fairness analysis; robustness is discussed mainly in relation to lighting & sink-material variation rather than user subgroups	Reflection & lighting sensitivity, dependence on motion for segmentation, prototype-stage deployment, & unclear large-scale real-world integration
[68]	Smartphone-based real-time guided handwashing assessment & behavioral feedback	Healthcare specialists, physicians, medical students, & other healthcare-trained participants	Explicit real-time audio-visual feedback with three modes: not guided, semi-guided, & fully guided step-by-step feedback	Strongest human-factors study in this batch: directly compares how different feedback designs shape user behavior & performance; smartphone chosen as familiar platform; no formal privacy or acceptability scale reported	smartphone video capture, live-stream or app-based evaluation workflow, operator/neural-network assessment pipeline, & potential data storage for all episodes; designed with future clinical integration in mind	No subgroup fairness analysis; participants were all healthcare-trained, so performance across broader staff groups or contexts was not assessed	Feedback effects may depend on interface design; underlying neural network was still under development & partly substituted by human operator; unclear generalizability to routine clinical use
[69]	Multi-view gesture-recognition benchmark	Medical staff / handwashing subjects recorded across 6 camera views & 6 locations	No real-time feedback implemented; focused on gesture-recognition benchmarking	Human factors not directly assessed; paper highlights realistic viewpoint mismatch & fine-grained action difficulty rather than usability	multi-camera deployment across multiple locations, large annotated dataset, viewpoint-specific train/test protocols, & models capable of cross-view generalization	Strong fairness/generalizability relevance: explicitly focuses on data distribution mismatch across viewpoints & imbalanced class distributions; recommends Macro F1-score because accuracy alone can be misleading	Cross-view distribution shift, class imbalance, & difficulty generalizing to new viewpoints/locations; primarily a benchmark/dataset paper rather than implementation study
[70]	Passive background surveillance & hospital-wide tracking linked to dispenser-use classification	Healthcare staff, patients, & visitors moving through hospital units; infection-control teams/managers are likely indirect users	Automated hand-hygiene event classification linked to tracked trajectories; intended to support compliance measurement & feedback, but no direct bedside alerting described	Privacy-focused design is central: uses de-identified depth images instead of RGB due to HIPAA/GDPR constraints; study emphasizes interpretability as a way to build trust with clinicians & patients	sparse network of depth sensors across units, pedestrian detection & cross-camera tracking, ground-truth annotation from multiple annotators, & depth-based activity classification linked to trajectories	No subgroup fairness analysis; study discuss challenges from crowded scenes, sparse overlapping views, & inability to use appearance cues because of privacy constraints	High infrastructure & annotation burden, crowded-scene tracking complexity, privacy-driven sensing constraints, & dependence on accurate linkage between room-entry events & dispenser use
[71]	Outpatient CV hand hygiene improvement project	Outpatient clinic staff/users; exact affected staff roles not fully visible in current snippet	Likely includes feedback or performance improvement component, but exact mechanism is not fully recoverable from the visible excerpt	Insufficient detail in current visible excerpt to characterize human-factors reporting confidently	Appears to be an applied outpatient-clinic implementation paper, but specific hardware/workflow requirements are not fully visible in the current excerpt	Insufficient detail from the visible excerpt to assess subgroup fairness or differential performance	Applied improvement setting suggested, but current visible excerpt is insufficient for precise extraction; row should be revisited if you want maximum accuracy
[72]	Real-time sink-side guidance & validation system	Hospital medical personnel, patients, & general users in public/private settings	Explicit real-time step-by-step guidance & completion notification on a display screen; validates each WHO step & threshold time	Human factors are described at a basic product level: low user expertise required, intended for broad public & clinical use, but no formal usability or acceptability evaluation was conducted	mounted camera above the wash basin, display screen, small processing unit, OpenCV-based motion/ROI extraction, trained DNN model, & developer-side installation/maintenance; system assumes one user in frame at a time	No subgroup fairness analysis; broad user classes are proposed, but performance across staff types, demographics, or varied real-world conditions was not evaluated	Prototype-stage system, reliance on fixed sink-top setup, potential multi-user-in-frame failure, controlled live-feed assumptions, & no real clinical deployment validation
[73]	Hybrid monitoring & behavior-change intervention with continuous automated monitoring plus additional modalities	Burn-center HCWs, including nurses, physicians, housekeeping personnel, & physical therapy/anesthesia technicians	Real-time badge color reminders (green/red), camera-based handwashing feedback at sinks, weekly individualized e-mail feedback, in-service training, & champion rewards in later phase	Stronger human-factors/implementation reporting than most studies: confidentiality guaranteed, voluntary participation, workflow described as non-disruptive, but badge use differed by profession & physician uptake decreased	zone sensors, soap/AHS dispenser motion sensors, individualized real-time reminder collar badges, camera modules above sinks, display screen, Wi-Fi-connected web dashboard, & sustained multimodal implementation	Differential performance across staff roles was directly observed: nurses, technicians, & housekeeping staff improved significantly earlier, whereas physicians showed weaker/less consistent response & reduced badge use; no formal fairness analysis beyond profession-specific comparisons	High infrastructure complexity, surveillance & badge dependence, profession-specific engagement differences, only 3 of WHO 5 moments captured, & sustainability depended on additional modalities
[74]	Hybrid IoT-integrated tracking & enforcement across hospital spaces using wearables, smart dispensers, & door control	Hospital personnel & patients wearing BLE-enabled devices in ICUs, inpatient rooms, elevators, & other facilities	No fully evaluated behavioral feedback study, but system tracks user ID/location, opens access workflow after sanitation, & supports web/mobile/cloud monitoring	Human factors are discussed mainly indirectly: goal is ease of use & future acceptability assessment; privacy/surveillance burden & wearable dependence are not empirically studied	BLE wearables, intelligent ABH dispensers, LoRaWAN gateway, Jetson Nano, cloud/database/mobile app stack, IR presence/proximity sensing, & hospital-wide network deployment	No subgroup fairness analysis; system depends on wearable carriage & human-presence verification logic, but differential performance across staff/patient groups was not assessed	Very high infrastructure complexity, wearable dependence, door/access-coupled enforcement, cloud/network maintenance burden, & limited evidence on real clinical acceptability or behavior change
[75]	Passive in-ward radar monitoring of hand-rub technique	HCWs performing alcohol-based hand rub inside wards	No direct user feedback in the paper; designed to monitor gesture sequence & timing unobtrusively at the dispenser	Privacy protection is a major explicit advantage: RF sensing is presented as more anonymous than depth/RGB cameras; designed for unattended in-ward use with lower privacy intrusion	mmWave radar mounted on/near dispenser, continuous RF sensing pipeline, weakly supervised sequence labelling, & training on gesture sequences collected from 10 subjects over 3 months	Important differential-performance/generalizability issues are explicitly discussed: mirrored gestures are harder to distinguish, real-life sequences deviate from ideal WHO order, segmentation quality strongly affects performance, & data were limited to 10 subjects	Real-world gesture variability, dependence on sequence-learning quality, limited subject pool, need for dispenser-mounted radar infrastructure, & no demonstrated large-scale hospital deployment
[76]	CCTV-based ICU recognition; future scoring/alerts	ICU clinicians/nurses recorded by existing CCTV cameras	No current live bedside alert system, but study position the system as a future scoring & alarming component of hospital IoT	Human factors not systematically assessed; major real-world constraints include side-view camera angle, foam occlusion, & motion blur; use of existing CCTV improves practical integration but not user-experience evidence	integration with existing ICU CCTV infrastructure, multi-view dataset collection, hand-landmark extraction, manual annotation, & subject-adaptive or cross-subject model improvement	Strong differential-performance signal: personalized models achieved about 95% accuracy whereas generalized models were only about 56%; the study attributes subject dependency to palm size & washing habits, & performance also varies by camera view & class imbalance	Subject-dependent performance, viewpoint variation, occlusion, motion blur, class imbalance, & limited cross-subject generalizability despite good personalized accuracy
[77]	Wearable-based background monitoring for in-hospital hand hygiene event, duration, & technique recognition	HCWs wearing wrist sensors in hospital settings	No direct real-time bedside feedback implemented in the reported work; sensor recording is triggered by dispenser messages & data are transferred for later analysis	Human factors not evaluated; wrist-worn approach is motivated as a way to overcome attribution problems in badge-based monitoring & avoid the framing restrictions of vision-only systems	sensor-equipped wrist devices on both wrists, dispenser-triggered radio messaging, gateway/base-station infrastructure, & sustained worker wear compliance	Strong differential-performance/generalizability relevance: experiments included unknown-subject testing, one-wrist vs two-wrist comparison, & multiple datasets; no formal fairness analysis across staff subgroups reported	Wearable dependence, need for triggering/network infrastructure, subject variability, & practical burden of continuous wrist instrumentation
[78]	Retrospective RGB-D pose recognition	Controlled handwashing subjects; not frontline deployment	No real-time user-facing feedback implemented; focused on pose recognition from video frames & clips	Human factors not directly assessed; relies on vertically projected camera view & controlled recording setup rather than real-use workflow assessment	RGB-D camera, hand segmentation using color-depth fusion, PCA/HOG feature extraction, & subject-independent cross-validation on a small controlled dataset	Subject-independent evaluation was performed with leave-one-person-out validation, but no formal subgroup fairness analysis was reported; performance may vary with camera/data quality, & color outperformed depth in this setup	Small controlled dataset, dependence on overhead camera alignment & segmentation quality, & unclear real-world deployment readiness
[79]	Real-time soap-lathering monitoring	Handwashing subjects at a basin; envisioned for health services & food preparation personnel	No deployed reminder; designed to warn after improper soap use	Human factors are discussed mainly as advantages over wearables: contactless design reduces contamination risk, battery burden, & misplacement; no formal acceptability study reported	fixed depth sensor mounted above basin, wireless trigger/ID infrastructure, low-power compute platform, fixed background/ROI setup, & gesture-sequence verification logic	No subgroup fairness analysis; evaluation used 15 subjects, & generalizability across broader staff/user groups & variable environments was not systematically assessed	Fixed basin-centered setup, trigger dependence, controlled environment assumptions, & limited participant diversity
[80]	Wearable-based continuous monitoring with zone-triggered reminders & server logging	Workers in hospitals, clinics, restaurants, & food businesses wearing smart watches	Explicit real-time reminders & quality alerts via vibration, beeps, & on-watch display; data also stored on a server	Human factors & deployment issues are discussed more directly than most studies: watch-based monitoring is presented as less privacy-intrusive than cameras & designed for scalable use, though uncomfortable environments from monitoring & acceptance are not formally quantified	smart watches, Bluetooth beacons, Bluetooth-enabled dispensers, relays/server infrastructure, adaptive scanning, & worker compliance with wearing devices	Person-dependent classification far outperformed person-independent classification	Wearable dependence, multiperson dispenser-zone ambiguity, infrastructure burden, battery/energy management, & weaker person-independent generalizability
[81]	Wearable-based background monitoring for duration & technique recognition, triggered by dispenser use	HCWs & study participants wearing wrist-worn commodity/custom sensors	No direct user feedback in the reported paper; dispenser-triggered sensing supports later classification of duration & technique	Human factors not evaluated; study frame wrist sensing as a way to attribute hand hygiene more accurately than proximity-only systems	wrist-worn accelerometer/gyroscope devices, dispenser-trigger messages, base-station/gateway setup, & possibly sensing on both wrists for better performance	Strong differential-performance/generalizability relevance: compares one wrist vs both wrists, accelerometer vs fused sensing, & reports leave-one-subject-out performance; no subgroup fairness analysis	Wearable dependence, subject variability, need for dispenser-linked infrastructure, & limited direct evidence on real-time clinical integration

Abbreviations: ABH, alcohol-based hand hygiene; ABHR, alcohol-based hand rub; AI, artificial intelligence; AVA, autonomous video auditing; AWS, Amazon Web Services; BLE, Bluetooth Low Energy; BiLSTM, bidirectional long short-term memory; CCTV, closed-circuit television; CNN, convolutional neural network; CTC, connectionist temporal classification; CV, computer vision; DNN, deep neural network; fps, frames per second; GDPR, General Data Protection Regulation; GUI, graphical user interface; HCWs, healthcare workers; HOG, histogram of oriented gradients; HIPAA, Health Insurance Portability and Accountability Act; ICU, intensive care unit; IMU, inertial measurement unit; IoT, Internet of Things; IR, infrared; LED, light-emitting diode; LoRaWAN, Long Range Wide Area Network; NFMI, near-field magnetic induction; OR, operating room; PCA, principal component analysis; PPE, personal protective equipment; RF, radio frequency; RFID, radio-frequency identification; RGB, red-green-blue; RGB-D, red-green-blue plus depth; ROI, region of interest; S3, Simple Storage Service; SSD, Single Shot MultiBox Detector; SVM, support vector machine; TensorRT, Tensor Runtime; VGG16, Visual Geometry Group 16-layer network; WHO, World Health Organization; Wi-Fi, wireless fidelity.

Across the included literature, workflow integration varied considerably. Some systems functioned as passive background surveillance tools, such as room-based computer vision systems, CCTV-supported monitoring, or wearable sensing platforms that recorded hand hygiene events without interrupting clinical tasks. Other systems were designed for real-time prompting or guided training, particularly sink-based vision systems and wearable reminder platforms that provided immediate visual, audio, or haptic feedback. A smaller group of studies described hybrid approaches that combined automated monitoring with team-level reports, individualized reminders, or broader institutional dashboards [35,60,73,80].

Human factors and implementation conditions were reported inconsistently. Some studies explicitly considered privacy-preserving design, reduced intrusiveness, hands-only capture, or anonymized sensing, while others discussed user interface preferences, participation patterns, or the burden associated with wearable devices [28,46,75,80]. However, most studies primarily emphasized technical performance and provided limited systematic evaluation of usability, acceptability, trust, alert burden, or perceived fairness.

Organizational readiness also emerged as an important but underreported dimension. Many systems depended on substantial infrastructure, including fixed camera positioning, multiple sensors, wearable devices, network connectivity, data pipelines, and setting-specific calibration [47,60,74,76]. Several studies suggested that deployment feasibility may be influenced by installation complexity, maintenance burden, interoperability with existing systems, and the need for local workflow adaptation.

In addition, Table 5 highlights that fairness-related analysis was generally limited. Formal subgroup fairness analysis was rarely reported, and only a small number of studies explicitly identified differential performance across users, staff groups, viewpoints, or environments [42,58,73,76]. Where such differences were described, they often involved poorer generalizability of user-independent models, reduced performance in uncontrolled real-world settings, or variation associated with camera angle, occlusion, or subject-specific movement patterns. Overall, these findings suggest that implementation feasibility, user experience, and performance heterogeneity remain incompletely addressed in the current evidence base.

## 4 Discussion

### 4.1 Core value and clinical significance of AI-based hand hygiene monitoring

#### 4.1.1 Overcoming limitations of traditional monitoring methods.

The integration of AI has the potential to address many of the inherent constraints of conventional hand hygiene monitoring. Compared with human observation, AI-based systems enable continuous and objective monitoring while minimizing Hawthorne-related distortion. Unlike human auditors whose physical presence triggers immediate behavioral changes, AI systems operate in the background. Nevertheless, AI systems may still be subject to a residual Hawthorne effect, because awareness of monitoring itself can influence behavior even in the absence of a human observer [82]. Consequently, AI systems may mitigate the Hawthorne effect rather than completely negate the psychological impact of surveillance. For instance, a study conducted in a burn center [73] demonstrated that AI-based automatic monitoring reported compliance rates approximately 12 percentage points lower than those obtained through manual observation, providing a more accurate representation of real-world practice.

Compared to indirect estimation methods relying on sanitizer consumption, AI systems can precisely identify “who, when, and where” missed hand hygiene opportunities, as demonstrated by a LoRaWAN-based system [74]. This capability allows infection control personnel to target interventions to specific individuals and contexts, shifting hand hygiene surveillance from retrospective review toward more proactive management.

#### 4.1.2 Enabling precision in hand hygiene management.

AI-based monitoring systems can detect hand hygiene events while also assessing procedural quality and standardization, addressing a key limitation of traditional methods that emphasize frequency over quality. For instance, one intelligent guidance system [72] identifies whether HCWs omit essential steps such as “rubbing fingertips” or “cleaning wrists.” Similarly, deep learning-based quality assessment research [51] has demonstrated the ability to generate procedural quality scores, improving the proportion of HCWs meeting both duration and quality standards from 45% to 72%.

Systems with real-time alerts can also notify users immediately when hand hygiene is missed. Some studies suggest higher compliance with real-time feedback than with delayed feedback. This shifts AI-based monitoring beyond documentation toward more active behavioral guidance.

#### 4.1.3 Tailoring surveillance to diverse clinical settings.

Based on the currently available evidence, different AI technical pathways may be better aligned with different clinical scenarios according to infection control needs, privacy requirements, mobility patterns, and infrastructure conditions. Fixed clinical areas such as operating rooms and ICUs may be more amenable to computer vision systems. One investigation [76] reported a hand hygiene event detection rate of 93.7% in a multi-bed ICU, suggesting potential utility in this specific setting for capturing transient behaviors among staff frequently entering and exiting patient zones.

For dynamic settings including outpatient and emergency departments, wearable sensor systems may offer a more adaptable option. In privacy-sensitive environments such as general wards or isolation rooms, radar/radio frequency-based systems [75] may offer a non-visual monitoring option based on limited currently available evidence. At the institutional level, IoT-integrated systems may facilitate more centralized monitoring and data aggregation under infrastructure-supported implementation conditions. For example, a LoRaWAN-based system [74] covering 100,000 square meters enabled unified data aggregation across multiple wards, providing continuous quantitative data for infection control decision-making.

#### 4.1.4 Interpreting technical performance in relation to clinical and implementation outcomes.

Although many included studies reported favorable technical metrics such as accuracy, sensitivity, or F1 score, these measures should not be interpreted as direct indicators of clinical effectiveness. In the context of hand hygiene monitoring, high algorithmic performance primarily demonstrates that a system can detect or classify predefined behaviors under specific validation conditions. By itself, it does not establish that the system reduces healthcare-associated infection burden or improves care quality. Nor does it demonstrate sustained behavior change over time or acceptability and feasibility for routine use by HCWs [83]. In the present review, most studies remained focused on technical development, controlled validation, or limited pilot deployment, whereas only a small number examined broader implementation issues such as workflow integration, user acceptability, or sustained compliance improvement [84,85]. Moreover, outcomes that are most meaningful for infection prevention practice, including long-term maintenance of behavioral change, integration with infection surveillance data, cost-effectiveness, and organizational impact, were rarely evaluated. Therefore, the current evidence base mainly supports technical feasibility and early implementation potential rather than established patient-centered or system-level benefit.

Nor is there sufficient evidence at present to show that AI-based monitoring provides superior infection prevention benefit, cost-effectiveness, or sustainability compared with well-implemented multimodal hand hygiene improvement programs [86]. Future research should more explicitly connect AI-based monitoring with pragmatic infection control outcomes, including longitudinal compliance trajectories, staff experience, unintended consequences, and linkage with infection surveillance data. In particular, AI-based nudge strategies may be useful for improving compliance, but their sustained effectiveness and contextual suitability still require evaluation in real-world clinical environments [85].

In addition, implementation studies should consider the possibility of automation bias, whereby infection control teams or frontline users may over-rely on imperfect AI outputs, especially when system benefits are perceived as high [87]. Reported accuracy and related performance measures should be interpreted in relation to validation context and evidence maturity. Metrics derived from technical development or laboratory validation studies mainly reflect performance under constrained conditions, whereas findings from pilot clinical evaluations suggest early implementation potential rather than robust evidence of generalizability or clinical readiness.

### 4.2 Key considerations for technical route selection

#### 4.2.1 Prioritizing scenario adaptability.

In scenarios characterized by varying risk levels, the selection of AI systems should be interpreted in light of contextual adaptability, based on the currently available evidence. For high-risk, stationary environments such as ICUs or operating rooms, computer vision systems may be more suitable, utilizing multi-angle camera coverage to mitigate occlusion effects. In highly mobile areas, such as outpatient clinics or emergency departments, lightweight wearable devices may be advantageous to balance mobility with recognition accuracy. In privacy-sensitive zones such as wards and treatment areas, radar/RF systems or privacy-preserving vision systems [28] may support monitoring while reducing information leakage risks.

#### 4.2.2 Balancing cost, effectiveness, and implementation burden.

Economic considerations remain insufficiently addressed in the current evidence base. Although some AI-based systems may reduce manual auditing workload or improve the timeliness of feedback, few studies formally evaluate whether these technologies are cost-effective relative to established multimodal hand hygiene improvement strategies [25,86]. The available evidence suggests that electronic or automated monitoring may offer operational advantages, but these benefits must be weighed against hardware costs, installation requirements, calibration, maintenance, staff training, and information technology support [25]. Accordingly, the practical value of AI systems should not be judged only by algorithmic performance or short-term compliance gains, but by whether they generate meaningful infection prevention benefit at an acceptable implementation cost. This issue is particularly important for low- and middle-income settings, where competing resource priorities may make high-cost monitoring platforms difficult to justify unless they demonstrably outperform lower-cost multimodal interventions [86,88].

#### 4.2.3 Compatibility with existing systems.

Compatibility of AI systems with existing hospital infrastructure and information systems may lower implementation barriers. If a closed-circuit television system is already operational [76], algorithmic modules may be added to existing camera systems, enabling low-cost upgrades. Similarly, if smart sanitizer dispensers are deployed [74], adding sensor modules can facilitate interoperability with the AI system, thereby obviating the need for redundant equipment procurement.

### 4.3 Existing challenges and future research directions

#### 4.3.1 Technological challenges and optimization strategies.

Despite their advantages in consistency and objectivity, the clinical implementation of AI systems remains constrained by several technical and operational barriers. Firstly, environmental adaptability and model generalizability remain limited. Variations in lighting conditions, camera angles, and individual motion patterns among HCWs can all compromise recognition accuracy. Future research should strengthen cross-institutional data sharing, federated learning, and standardized motion databases to improve robustness across clinical settings. For example, multiple hospitals could collaborate to develop a common hand hygiene motion database based on standardized annotation protocols, shared definitions of hand hygiene opportunities and procedural steps, and consistent metadata on clinical setting, camera position, and participant role. Under a federated learning framework, each institution could retain raw video or sensor data locally while sharing only model parameters or encrypted updates [89,90]. This would allow algorithms to be trained across heterogeneous sites without centralizing sensitive behavioral data. Such an approach may improve cross-site generalizability while also supporting privacy protection and governance compliance.

#### 4.3.2 Practical implementation, human factors, and organizational readiness.

Beyond technical performance, the findings of this review suggest that successful implementation of AI-based hand hygiene monitoring systems is likely to depend on workflow fit, user experience, and organizational conditions. As summarized in Table 5, included systems differed substantially in how they were embedded into practice, ranging from passive background surveillance and retrospective review to real-time prompting, sink-based guided training, wearable reminders, and hybrid monitoring-feedback architectures [30,35,45,73]. These different integration modes are likely to have distinct implications for workflow disruption, behavioral immediacy, and operational burden.

Human factors were addressed unevenly across the literature. A subset of studies reported privacy-preserving designs, anonymized sensing, interface preferences, or practical issues related to wearability and device placement [28,46,75,80]. However, most studies did not systematically evaluate user acceptance, trust, perceived fairness, alert burden, or usability in routine clinical work. This gap is important because even technically accurate systems may fail if they are experienced as intrusive, disruptive, difficult to use, or poorly aligned with frontline workflow.

Organizational readiness was also seldom examined directly. Many systems required substantial local infrastructure, including multiple cameras, sink-mounted devices, wearable tags, wireless communication systems, and continuous calibration or maintenance [49,60,74,76]. These findings suggest that implementation should not be understood solely as a technical problem, but also as an organizational one involving governance, training, workflow redesign, IT support, and long-term operational sustainability.

Taken together, these findings indicate that future evaluations should move beyond technical feasibility alone and examine whether AI-based hand hygiene systems can be integrated into routine practice in ways that are acceptable to users, operationally sustainable, and responsive to the realities of different healthcare settings.

These challenges may be especially pronounced in low-resource settings. AI-based hand hygiene monitoring systems often presuppose several enabling conditions [88]. These include reliable electricity supply, stable network connectivity, routine equipment maintenance, procurement pathways for replacement parts, locally available technical support, and sufficient digital literacy among end users and managers. In settings where infection prevention teams are already understaffed and essential hand hygiene resources remain inconsistently available, introducing complex AI infrastructures may be less feasible than strengthening basic multimodal hand hygiene programs [86,88]. Therefore, the absence of evidence from low-income countries should not be interpreted simply as a research gap, but also as a signal that the infrastructural and workforce assumptions underlying many AI systems may not yet be transferable across settings. Future research should therefore incorporate implementation-science approaches to evaluate adoption, acceptability, feasibility, fidelity, sustainability, and context-specific barriers alongside technical performance.

#### 4.3.3 Ethical and institutional considerations of AI-based monitoring.

In addition, the current evidence base provides only limited insight into algorithmic bias and differential performance across users or contexts. Across the included studies, formal subgroup fairness analysis was rarely reported. Nevertheless, several studies identified subject-dependent or context-dependent variation, including weaker performance of generalized compared with personalized models, reduced accuracy under different camera viewpoints or occlusion conditions, and lower robustness in uncontrolled real-world settings [42,54,76]. These findings suggest that apparently strong aggregate performance metrics may obscure uneven system behavior across staff roles, environments, or user characteristics.

These systems may collect or infer worker-linked behavioral data through video, location, wearable, or event-log signals. Therefore, the ethical issues extend beyond privacy alone and should also be considered in relation to data governance and applicable legal frameworks. In settings where staff can be directly or indirectly identified, such data may fall within the scope of data protection laws such as the General Data Protection Regulation (GDPR). Where monitoring data are linked with identifiable health information handled by covered entities or business associates, obligations under the Health Insurance Portability and Accountability Act (HIPAA) may also become relevant. Accordingly, future implementations should clearly specify what data are collected, for what purpose, and who can access them [91–93]. They should also define how long the data are retained, whether they are de-identified, and whether they may be used only for quality improvement rather than punitive performance management. Broader healthcare AI literature similarly notes that acceptable overall model performance may coexist with biased or uneven behavior driven by data imbalance, measurement bias, and context-specific model development [94]. Future studies should therefore report subgroup-stratified performance, for example across staff roles, care settings, camera viewpoints, and user-dependent versus user-independent workflows, using measures such as sensitivity, specificity, false-alarm rates, and calibration rather than overall accuracy alone [95].

The implications of surveillance-based monitoring systems for workplace culture also warrant closer attention. Although these technologies are often framed as tools for supportive quality improvement, continuous monitoring may also be perceived as managerial surveillance if governance boundaries are unclear or if data are repurposed for punitive evaluation. In practice, this implies the need for explicit governance safeguards. These may include staff consultation before deployment, role-based access control, audit trails, clear retention limits, and restrictions on the secondary use of monitoring data. Such dynamics may erode trust, reduce psychological safety, and weaken healthcare worker engagement with infection prevention efforts. Qualitative research on video-based hand hygiene monitoring has similarly suggested that HCWs’ responses to surveillance-oriented systems are context-dependent and may be shaped by concerns about punitive consequences, confidentiality, data security, patient privacy, and the way feedback is delivered [96].

Automation bias represents another underexamined implementation risk. Infection prevention teams or local managers may over-rely on AI-generated dashboards, compliance summaries, or alerts, particularly when outputs are framed as objective or precise. However, imperfect classification, context-insensitive outputs, and limited generalizability mean that these systems should be interpreted as decision-support tools rather than substitutes for professional judgment, contextual review, or local infection prevention expertise. This concern has also been raised more broadly in clinical AI implementation, where assistive systems may foster misplaced confidence and reduce critical oversight [97].

Finally, the effects of feedback mechanisms are unlikely to be uniform across institutions. Real-time reminders, individualized alerts, or team-level reports may function differently depending on local safety climate, leadership framing, and staff trust in monitoring processes. In supportive, learning-oriented environments, feedback may reinforce improvement; in punitive or low-trust settings, the same feedback mechanisms may provoke resistance, disengagement, or performative compliance. Recent hand hygiene research agendas have specifically identified institutional safety climate as a key determinant and priority area for understanding how feedback and improvement strategies translate into sustained hand hygiene performance [10].

For this reason, future implementation research should evaluate not only technical validity, but also fairness, accountability, organizational context, and the unintended consequences of AI-based monitoring in routine clinical environments.

### 4.4 Limitations

Several limitations should be considered when interpreting the findings of this review. First, as a scoping review, it did not include formal critical appraisal or risk-of-bias assessment of the included studies. Second, substantial heterogeneity exists across the included literature with respect to technical methodologies, study designs, outcome definitions, reference standards, validation strategies, and reported performance measures, which limits direct comparison across studies. In addition, implementation-related dimensions such as workflow integration, human factors, organizational readiness, subgroup fairness, and automation-related risks were inconsistently reported across the included studies. As a result, these issues could be synthesized qualitatively, but not compared systematically across technologies, settings, or user groups. Third, many included studies were conducted in controlled environments, simulated settings, single institutions, or small pilot samples, which may limit generalizability to broader healthcare contexts. Finally, some important implementation factors, including workplace culture, staff trust, institutional safety climate, and the unintended consequences of surveillance-oriented monitoring, were only indirectly addressed in the available literature. Therefore, the findings should be interpreted primarily as an evidence map of the current field rather than as a basis for definitive conclusions regarding comparative effectiveness or implementation success.

## 5 Conclusion

AI-based technologies offer a promising and evolving approach to support hand hygiene monitoring in healthcare settings. Across computer vision, wearable sensor, radar/radio frequency-based, and IoT-integrated systems, existing studies suggest that these technologies can support automated hand hygiene monitoring. Reported functions include event detection, action recognition, duration assessment, and, in some cases, real-time feedback. However, the current evidence base is still dominated by small-scale technical validation studies and limited pilot deployments, with insufficient evidence regarding long-term reliability, cost-effectiveness, workflow integration, staff acceptability, and broader real-world implementation. Importantly, favorable technical performance should not be interpreted as equivalent to demonstrated clinical effectiveness. At present, the available evidence more strongly supports technical feasibility than patient-centered or system-level benefit, and the field remains at a largely pre-translational stage. Future research should therefore prioritize pragmatic trials and standardized reporting and evaluation frameworks for AI hand hygiene systems. It should also examine integration with infection surveillance data, implementation-science outcomes, unintended consequences, and long-term sustainability in routine practice.

## Supporting information

S1 Appendix PRISMA-ScR checklist. Preferred reporting items for systematic reviews and meta-analyses extension for scoping reviews checklist.
(DOCX)

S2 Appendix Search strategies. Detailed search strings and strategies for all searched databases.
(PDF)

S3 Appendix Extracted data from included studies. The standardized data extraction form containing study characteristics, technical features, and performance metrics of all 45 included studies.
(XLSX)

S4 Appendix Classification and coding framework. The structured framework used for the thematic analysis of AI technological pathways and implementation challenges.
(DOCX)

S5 Appendix Full-text screening and exclusion reasons. A complete list of studies excluded at the full-text screening stage with specific reasons for exclusion.
(XLSX)

## Abbreviations

AI: Artificial Intelligence
IoT: Internet of Things
ICU: Intensive Care Unit
JBI: Joanna Briggs Institute
LoRaWAN: Long Range Wide Area Network
PCC: Population, Concept, Context
PRISMA-ScR: Preferred Reporting Items for Systematic Reviews and Meta-Analyses Extension for Scoping Reviews
RF: Radio Frequency
RGB: Red-Green-Blue
WHO: World Health Organization
