# Supplementary material for: Artificial intelligence for monitoring hand hygiene compliance in healthcare settings: A scoping review
Source: PLoS One. 2026 Apr 21;21(4):e0347683. doi: 10.1371/journal.pone.0347683 (PMC13098956; doi:10.1371/journal.pone.0347683)
Supplement: S2 Appendix — Detailed search strings and strategies for all searched databases. (PDF) [file pone.0347683.s002.pdf]

S2 Appendix: Search Strategy.

Database	Search terms	Results
Pubmed	("Hand Hygiene"[Mesh] OR "Hand Disinfection"[Mesh] OR hand hygiene[tiab] OR hand disinfection[tiab] OR handwashing[tiab] OR "hand washing"[tiab] OR hand sanitization[tiab]) AND ("Artificial Intelligence"[Mesh] OR "Machine Learning"[Mesh] OR "Deep Learning"[Mesh] OR "Neural Networks, Computer"[Mesh] OR "Computer Vision Systems"[Mesh] OR AI[tiab] OR artificial intelligence[tiab] OR machine learning[tiab] OR deep learning[tiab] OR computer vision[tiab] OR neural network[tiab] OR intelligent system[tiab])	79
Scopus	(TITLE-ABS-KEY (("Hand Hygiene" OR "Hand Disinfection" OR handwash* OR "hand wash*" OR "hand sanitiz*" OR "hand sanitisation"))) AND (TITLE-ABS-KEY (("Artificial Intelligence" OR "Machine Learning" OR "Deep Learning" OR "Computer Vision" OR "Neural Network*" OR AI OR "intelligent system*" OR "convolutional neural network*" OR cnn OR "recurrent neural network*" OR rnn OR "object detection" OR "image processing" OR "video analysis"))) AND (TITLE-ABS-KEY ((health* OR hospital* OR "medical" OR "clinical" OR "patient" OR "health care" OR healthcare OR "intensive care unit" OR ICU OR "ward" OR "healthcare setting" OR "healthcare facility" OR "clinical environment")))	280
Embase	(('hand hygiene'/exp OR 'hand disinfection'/exp OR (hand NEXT/3 (hygiene OR disinfect* OR wash* OR sanitiz*)):ti,ab,kw) AND ('artificial intelligence'/exp OR 'machine learning'/exp OR 'deep learning'/exp OR 'neural network'/exp OR (ai OR 'artificial intelligence' OR 'machine learning' OR 'deep learning' OR 'computer vision' OR 'convolutional neural network*' OR cnn OR 'recurrent neural network*' OR rnn OR 'object detection' OR 'image processing' OR 'video analysis'):ti,ab,kw) AND ('health care'/exp OR hospital/exp OR 'hospital ward'/exp OR 'intensive care unit'/exp OR (health* OR hospital* OR clinic* OR inpatient* OR outpatient* OR 'health care' OR healthcare OR 'intensive care unit' OR ICU OR ward OR department):ti,ab,kw))	180
Web of Science	TS=(("hand hygiene" OR "hand disinfection" OR handwash* OR "hand wash*" OR "hand sanitiz*") AND ("artificial intelligence" OR "machine learning" OR "deep learning" OR "computer vision" OR "neural network*" OR "convolutional neural network*" OR CNN OR "object detection" OR "image processing" OR "video analysis") AND (hospital* OR "health care" OR healthcare OR clinic* OR "intensive care unit" OR ICU OR ward OR "healthcare setting" OR "clinical environment"))	100
IEEE Xplore	("Hand Hygiene" OR "Hand Disinfection" OR handwash* OR "hand wash*" OR "hand sanitiz*") AND ("Artificial Intelligence" OR "Machine Learning" OR "Deep Learning" OR "Computer Vision" OR "Neural Network*" OR AI OR "intelligent system*" OR "convolutional neural network*" OR CNN OR "recurrent neural network*" OR RNN OR "object detection" OR "image processing" OR "video analysis" OR YOLO) AND (hospital OR "health care" OR healthcare OR clinic OR "intensive care unit" OR ICU OR ward OR "healthcare setting" OR "clinical environment")	86
