# Supplementary material for: Artificial intelligence for monitoring hand hygiene compliance in healthcare settings: A scoping review
Source: PLoS One. 2026 Apr 21;21(4):e0347683. doi: 10.1371/journal.pone.0347683 (PMC13098956; doi:10.1371/journal.pone.0347683)
Supplement: S4 Appendix — The structured framework used for the thematic analysis of AI technological pathways and implementation challenges. (DOCX) [file pone.0347683.s004.docx]

**S4 Appendix: Classification and Coding Framework.**

| **Domain** | **Variable name** | **Operational definition** | **Data type** | **Coding options / allowable values** | **Notes for coders** |
| --- | --- | --- | --- | --- | --- |
| A. Bibliographic information | study_id | Unique identifier assigned to each included study in the shared dataset. | Text | S01-S45 | Create a stable study code for cross-referencing all supplementary files. |
| A. Bibliographic information | reference_no | Reference number used in the manuscript tables. | Text | [27], [30], ... | Keep identical to manuscript numbering to ensure traceability. |
| A. Bibliographic information | full_citation | Full bibliographic citation of the included article. | Text | Free text | Use the final accepted reference format. |
| A. Bibliographic information | publication_year | Year of publication. | Numeric | 2007-2025 | Record as four digits. |
| A. Bibliographic information | country_region | Country or region where the study was conducted. | Categorical | Single country / Multi-country / Not reported | Add country names in a companion text field when reported. |
| A. Bibliographic information | study_design | Primary study design reported by the authors. | Categorical | Experimental / Quasi-experimental / Technical validation / Diagnostic accuracy / Other | Use the categories already summarized in Table 2 of the manuscript. |
| B. Study context and maturity | evaluation_type | Maturity level of system evaluation. | Categorical | Laboratory test / Simulated clinical scenario / Pilot deployment / Real-world deployment | Added in response to reviewers requesting differentiation by evidence maturity. |
| B. Study context and maturity | setting_type | Clinical or simulated environment in which the system was evaluated. | Categorical | General ward / ICU / Operating room / Emergency department / Outpatient area / Mixed / Simulated healthcare setting / Other | Use the most specific healthcare setting reported. |
| B. Study context and maturity | real_world_status | Whether the study was performed in an actual clinical workflow. | Binary | 0 = simulated or laboratory; 1 = real-world clinical deployment | Useful for stratified synthesis. |
| B. Study context and maturity | participant_type | Population involved in the study. | Categorical | Healthcare workers / Simulated participants / Mixed / Not reported | Align with the PCC-based eligibility criteria. |
| B. Study context and maturity | sample_size | Number of participants, observations, clips, events, or sessions used for evaluation. | Text | Numeric or author-reported value | Retain author wording when the unit is not directly comparable across studies. |
| B. Study context and maturity | study_duration | Observation or deployment period. | Text | Free text | Record exact duration where available (e.g., 2 weeks, 6 months). |
| C. AI system characteristics | technology_route | Main technological pathway of the monitoring system. | Categorical | Computer vision-based / Wearable sensor-based / Radar or RF-based / IoT-integrated AI / Hybrid-other | Use the four core routes synthesized in the Results section. |
| C. AI system characteristics | hardware_modality | Primary sensing hardware used to capture hand hygiene behavior. | Multiple choice | RGB camera / Depth camera / Thermal camera / Wearable IMU / RFID / Radar / Wi-Fi / Smart dispenser / Multi-sensor / Other | Code all applicable modalities. |
| C. AI system characteristics | algorithm_architecture | Main AI algorithm or model architecture. | Text | CNN / 3D CNN / LSTM / Pose estimation / Transfer learning / Rule-based ML / Other | Use author terminology; normalize obvious synonyms where possible. |
| C. AI system characteristics | privacy_preserving_design | Technical privacy protections incorporated into the system. | Multiple choice | None reported / Skeletonization / De-identification / Edge processing / Local storage only / Access control / Other | Added to support the ethics and governance discussion. |
| C. AI system characteristics | feedback_capability | Whether the system provides prompts or nudges to influence behavior. | Categorical | None / Real-time alert / Delayed feedback dashboard / Both | Include AI-based nudges when reported, per reviewer request. |
| D. Monitoring target and functions | hh_target | Hand hygiene target concept monitored by the system. | Categorical | WHO Five Moments / Room entry-exit opportunities / Handwashing steps / Dispenser use / Procedure-specific indication / Other | Choose the dominant target defined by the study. |
| D. Monitoring target and functions | core_function | Primary monitoring or analytic function of the system. | Multiple choice | Event detection / Action recognition / Duration assessment / Compliance classification / Staff tracking / Dashboard analytics / Other | Code all functions explicitly evaluated. |
| D. Monitoring target and functions | identification_level | Granularity of detection output. | Categorical | Individual-level / Group-level / Anonymous aggregate / Not clear | Important for linking technical capability with surveillance implications. |
| D. Monitoring target and functions | reference_standard | Comparator used to judge system performance. | Categorical | Human direct observation / Expert annotation / Sensor benchmark / System log / Not reported | Needed because reviewer comments highlighted heterogeneity in validation reference standards. |
| E. Validation and performance | validation_strategy | Approach used to validate model or system performance. | Categorical | Internal split / Cross-validation / External validation / Prospective field test / Not reported | Do not infer external validation unless explicitly stated. |
| E. Validation and performance | performance_metrics | Metrics reported for technical evaluation. | Multiple choice | Accuracy / Sensitivity or Recall / Specificity / Precision / F1-score / AUC / Kappa / Agreement / Time-to-detection / Other | Mark all reported metrics; do not force absent metrics. |
| E. Validation and performance | key_results | Main quantitative performance findings reported by the study. | Text | Free text | Keep as author-reported values; avoid recalculation across heterogeneous metrics. |
| E. Validation and performance | comparability_note | Flag indicating whether results are directly comparable with other studies. | Categorical | Limited comparability / Moderately comparable / Broadly comparable | Recommended because reviewers noted that accuracy percentages should not be over-interpreted. |
| E. Validation and performance | workflow_integration | Extent to which the system was integrated into routine infection prevention workflow. | Categorical | Standalone prototype / Partial integration / Routine workflow integration / Not reported | Supports a more implementation-oriented synthesis. |
| F. Human factors, ethics, and implementation | human_factors_issue | Human-factor themes reported by authors. | Multiple choice | Usability / Acceptability / Alert fatigue / Workflow burden / Training need / Safety climate / Not reported / Other | Added in response to reviewers asking for stronger human factors synthesis. |
| F. Human factors, ethics, and implementation | implementation_barriers | Barriers to adoption or performance reported in the study. | Multiple choice | Technical reliability / Occlusion / Multi-person interference / Cost / Infrastructure / Data quality / Staff resistance / Maintenance burden / Other | Code all explicitly described barriers. |
| F. Human factors, ethics, and implementation | ethical_governance_issue | Ethical or governance concern discussed in the article. | Multiple choice | Privacy / Consent / Data security / Algorithmic bias or fairness / Surveillance culture / Legal compliance / Not reported / Other | Added to operationalize reviewer requests regarding GDPR/HIPAA, fairness, and surveillance concerns. |
| G. Outcomes beyond technical accuracy | behavioral_outcome | Whether the study assessed change in hand hygiene behavior or compliance over time. | Categorical | Improved / No clear change / Measured without pre-post comparison / Not assessed | Separate from technical detection accuracy. |
| G. Outcomes beyond technical accuracy | clinical_outcome | Whether downstream infection-related or patient-care outcomes were assessed. | Categorical | HAI outcome reported / Proxy quality outcome only / Not assessed | Added because reviewers requested linkage to patient- and system-level outcomes. |
| G. Outcomes beyond technical accuracy | economic_outcome | Whether cost, cost-effectiveness, or resource implications were reported. | Categorical | Reported quantitatively / Discussed qualitatively only / Not reported | Useful for feasibility and LMIC relevance. |
| G. Outcomes beyond technical accuracy | future_direction | Main future research or implementation priorities proposed by the authors. | Text | Free text | Can later be thematically grouped (e.g., standardization, interoperability, federated learning). |
